# Single cell and spatial transcriptomic profiling of the type 2 diabetic coronary microcirculation and myocardium

**DOI:** 10.1007/s00395-025-01144-7

**Published:** 2025-11-07

**Authors:** Patricia E. McCallinhart, Corinne H. Strawser, Elizabeth A. R. Garfinkle, Jaye B. Navarro, Cynthia McAllister, Tatyana A. Vetter, Pamela A. Lucchesi, Elaine R. Mardis, Louisa Mezache, Rengasayee Veeraraghavan, Katherine E. Miller, Aaron J. Trask

**Affiliations:** 1 Center for Cardiovascular Research and The Heart Center, The Abigail Wexner Research Institute at Nationwide Children’s Hospital, Columbus, OH 43205 USA; 2 Steve and Cindy Rasmussen Institute for Genomic Medicine, The Abigail Wexner Research Institute at Nationwide Children’s Hospital, Columbus, OH 43205 USA; 3 Morphology Core Laboratory, The Abigail Wexner Research Institute at Nationwide Children’s Hospital, Columbus, OH 43205 USA; 4 Microscopy Core Laboratory, The Abigail Wexner Research Institute at Nationwide Children’s Hospital, Columbus, OH 43205 USA; 5 Center for Gene Therapy, The Abigail Wexner Research Institute at Nationwide Children’s Hospital, Columbus, OH 43205 USA; 6 Department of Pediatrics, College of Medicine, The Ohio State University Wexner Medical Center, Columbus, OH USA; 7 Department of Undergraduate Medical Education, University of Texas Tyler School of Medicine, Tyler, TX USA; 8 Department of Biomedical Engineering, The Ohio State University, Columbus, OH USA

**Keywords:** Coronary microvascular disease, Spatial transcriptomics, Diabetes, Single cell RNA sequencing, Cardiac

## Abstract

Coronary microvascular disease (CMD) is an early complication of type 2 diabetes (T2D) involving adverse endothelial and smooth muscle function, vascular remodeling, and alterations in mechanics. These culminate in impaired coronary blood flow. To interrogate transcriptional differences potentially contributing to CMD, we tested the hypothesis that comprehensive single-cell and spatial transcriptomic profiling of the coronary microcirculation and surrounding myocardium will identify new pathways to target in CMD. We utilized an innovative combination of single-cell RNA profiling and spatial transcriptomics to examine transcriptional differences and molecular signatures of CMD in T2D mice. Single-cell RNA profiling and spatial transcriptomics revealed an upregulation of genes linked to adipogenesis, fatty acid metabolism, and oxidative phosphorylation in T2D cell clusters and coronary microvascular-enriched regions. In ECs, VSMCs, cardiomyocyte clusters, fibroblasts, and macrophages, the upregulation of adipogenesis was directed by *Angplt4* and *Ephx2*, whereas *Hmgcs2* and *Acot2* were the key players in the upregulation of fatty acid metabolism, and *Pdk4* and *Ech1* were the drivers of oxidative phosphorylation upregulation. These intriguing data support the well-documented concept that cardiac metabolic inflexibility in T2D heart failure—characterized by reduced mitochondrial function, increased reliance on fatty acid oxidation, and impaired glucose utilization—contributes to oxidative stress and lipotoxicity. Our data unveiled novel and unique gene expression signatures of coronary microvessels in the presence and absence of diabetes.

**Supplementary Information:**

The online version contains supplementary material available at 10.1007/s00395-025-01144-7.

## Introduction

Cardiovascular disease (CVD) is the leading cause of death in type 2 diabetes (T2D) and has been associated with up to fourfold higher mortality [12, 53]. Over 14% of adults in the U.S. have T2D, and that number continues to rise at a rapidly alarming rate [1]. In both T2D experimental models and human patients, alterations in vessel structure and function result in micro- and macro-vasculopathies, which underlie many diabetic cardiovascular complications, including retinopathy, nephropathy, coronary artery disease, myocardial infarction, and stroke [8]. Recent studies have shown that coronary microvascular disease (CMD) independently predicts cardiac morbidity and mortality in obesity and metabolic disease, with coronary flow reserve (CFR) outperforming body mass index (BMI) and other risk factors in risk prediction [42].

CMD manifests due to endothelial cell- and vascular smooth muscle cell (VSMC)-dysfunction and alterations in remodeling and mechanics of the coronary resistance microvascular (CRM) wall that ultimately culminates in impaired coronary blood flow (CBF) [33, 42]. CRMs regulate CBF, and studies have demonstrated that T2D reduced coronary endothelial-mediated vasodilatory responses and augmented myogenic tone [42]. In addition, previous studies from our laboratory have demonstrated inward hypertrophic remodeling in T2D mice and metabolic syndrome (MetS) pigs that is associated with decreased lumen diameter, increased wall thickness, and reduced VSMC stiffness, but not endothelial stiffness [30, 43, 65]. Moreover, our studies have also shown that the 40–45% reduction in CBF in diabetic animals is consistent with both experimental data based on passive radius changes and standard flow resistance models, suggesting that remodeling and mechanics play a role in the reduction of CBF, and therefore, heart disease, during diabetes [30, 44, 65]. These data suggest that cell- and vascular layer-specific mechanisms are responsible for CMD. It is important to recognize that changes in the coronary microcirculation occur within the context of a dynamic myocardium, and therefore changes in myocardial function may influence the ability of CRMs to regulate CBF. To interrogate transcriptional differences potentially contributing to CMD, we tested the hypothesis that comprehensive single-cell and spatial transcriptomic profiling of the coronary microcirculation, perivascular region, and surrounding myocardium will identify new pathways to target in CMD.

## Methods

### Reagents

Antibodies and stains for immunostaining of tissue: anti-Cx43 (Sigma C6219), α-smooth muscle actin (Invitrogen 53–9760-82), DAPI (MP Biomedical 157,574), and Wheat germ agglutin, WGA, (Invitrogen W11262).

### Data availability

All sequencing data presented in this publication has been deposited in NCBI’s Gene Expression Omnibus (GEO; scRNA-seq: GSE290095; Visium: GSE290094) with a release date of 12/31/2026.

### Code availability

All code associated with the analyses presented in this study are available at https://github.com/miller-genomics-lab/trask_murine_t2d_spatial_transcriptomics.

### Animal model

This study was conducted in accordance with the National Institutes of Health Guidelines, and it was approved by the Institutional Animal Care and Use Committees at The Ohio State University and Nationwide Children’s Hospital. Male T2D homozygous db/db and control non-diabetic heterozygous Db/db were obtained from The Jackson Laboratories. The db/db mice are leptin receptor deficient and develop obesity, hyperglycemia, insulin resistance, and dyslipidemia by 4–8 weeks of age. They have documented CMD, nearly identical to that observed in the preclinical Ossabaw porcine model of metabolic syndrome, as evidenced by enhanced myogenic tone, impaired vasodilatory capacity, inward hypertrophic remodeling, and reduced CBF [65]. Importantly, rodents are resistant to atherosclerosis unless crossed with apolipoprotein E (ApoE) or low-density lipoprotein (LDL) receptor knockouts, which makes the utilization of db/db mice an excellent model for studying CMD independent of atherosclerosis [51]. Mice were housed under a 12-h light/dark cycle at 22 °C and 60% humidity and were allowed ad libitum access to standard low-fat laboratory chow and water. All experiments were conducted at 16 weeks of age corresponding to the presence of established CMD as previously described by us [30, 43]. These mice also display clinical indices of heart failure with preserved ejection fraction (HFpEF) [55, 57, 60].

### Fixed single cell RNA profiling (scRNA-seq)

Using aseptic, RNA-preserving conditions, control Db/db and T2D db/db mouse hearts were gravity-fed perfusion fixed under deep isoflurane anesthesia (3%) by ~ 15 s of a PBS flush, followed by ~ 15 s of 4% paraformaldehyde (PFA), and finished with 4% PFA with 60 mM KCl until cardiac arrest in diastole. Tissues were fixed for 24 h in 4% PFA at 4 °C and stored in 70% ethanol until paraffin embedding. Six 50 µm scrolls were cut from one control Db/db and one diabetic db/db formalin-fixed and paraffin-embedded (FFPE) heart blocks. Cells were isolated from scrolls by following the *Isolation of Cells from FFPE Tissue Sections for Chromium Fixed RNA Profiling* protocol from 10X Genomics (CG000632 RevB) using the gentleMACS Octo Dissociator method. Approximately 200,000 isolated cells for each sample were used as input for the Chromium Fixed RNA Profiling kit from 10X Genomics (CG000477 RevD) according to the manufacturer’s protocol. Briefly, cells were hybridized overnight with mouse BC001 probe set (Singleplex, ThermoFisher Scientific), followed by GEM generation and barcoding, and Illumina compatible library preparation. Libraries were sequenced on the Singular Genomics G4 benchtop sequencer; therefore, the Illumina libraries were converted using Singular Genomics conversion PCR primers. We targeted 25,000 read pairs per 10,000 cells for each sample and used a run recipe of: 28 × 10 × 10 × 50 (read1:i7index:i5index:read2). Demultiplexed FASTQ files were generated on-board the Singular G4 sequencer. Alignment to the Chromium Mouse Transcriptome Probe Set v1.0.1, filtering, barcode counting, and UMI counting was performed using Cell Ranger multi v7.1.0 following the default parameters.

### 10X Genomics Visium spatial transcriptomic assay

Paraffin-embedded tissue blocks were sliced into 5 µm sections and transferred to the 6.5 mm × 6.5 mm capture areas on Visium slides according to the 10X Genomics demonstrated protocol (CG000408). Prior to imaging, FFPE tissue mounted on Visium slides was deparaffinized, decrosslinked, and immunofluorescent stained (Cx43 1:50 from Sigma-Aldrich (St. Louis, MO), secondary 647 1:400 from Thermo Fisher Scientific (Grand Island, NY), Wheat Germ Agglutin 1:250 from Invitrogen, 594 conjugate (Eugene, OR), Alpha-SMA direct 488 conjugate 1:250 from Invitrogen (Eugene, OR), and DAPI 1:250 from MP Biomedicals (Solon, OH) according to the demonstrated protocol from 10× Genomics (CG000410). Slides were incubated overnight at 60 °C prior to deparaffinization, followed by sequential immersion in xylene for 10 min (2 times), 100% ethanol for 3 min (3 times), 95% ethanol for 3 min (2 times), 85% ethanol for 3 min, 70% ethanol for 3 min, and Milli-Q water for 20 s. The slides were then washed twice with Tris–EDTA (TE) buffer and incubated for 1 h at 70 °C to decrosslink the deparaffinized tissue.

Next, the sections were blocked for 5 min using bovine serum albumin (Millipore Sigma, 126,615-25ML), followed by 1 h incubation with cx43 primary antibody (1:50 dilution; Sigma) at room temperature. The Visium slides were then washed three times with PBS-tween and then subjected to a 45-min incubation with the secondary antibody 647 (1:400; ThermoFisher), Wheat Germ Agglutin 568 (1:250; Invitrogen), direct-conjugate alpha-smooth muscle actin antibody 488 (1:250; Invitrogen), and DAPI (1:250 dilution; MP Biomedical). Next, the slides were washed three times with PBS-tween followed by a final PBS wash before being cover slipped. Multichannel fluorescence images of the whole-heart sections and fiducial frame were captured on a Nikon Ti2-E inverted microscope with a Nikon D-LEDI multiline LED engine and a Hamamatsu ORCA Fusion sCMOS camera (16-bit mode). NIS-Elements AR software (v5.3) with the JOBS module was used for configuring automated acquisition. A high-speed filter wheel with single-channel filters for DAPI, GFP, TRITC/Cy3, and Cy5 was used for emission signal cleanup. Images were captured using a 10 × Nikon Plan Apochromat Lambda D objective for a final resolution of 0.65 µm/pixel. The fiducial frame was captured using the TRITC/Cy3 LED and filter with a 500 ms exposure time to ensure adequate detection in addition to the four channels optimized for tissue. The fiducial channel was processed with rolling ball background subtraction when stitched, and all other channels were stitched without further processing.

After imaging, sample tissue was prepared using the demonstrated protocol from 10× Genomics (CG000407). Sample tissues were permeabilized for 15 min at room temperature followed by the addition of mouse whole transcriptome probes (part numbers 2000457 for RHS probes and 2,000,458 for LHS probes) and hybridization for 18 h at 50 °C using the 10X Visium Cassette and 10X Thermalcycler Adapter. Samples were then washed with 50 °C pre-heated FFPE Post Hybridization Wash Buffer (2,000,424), followed by three sequential washes with 50 °C 2× concentrated saline-sodium citrate buffer. The slide was then allowed to cool to room temperature before adding Probe Ligation Mix to ligate the Right-Hand Side (RHS) and Left-Hand Side (LHS) probes together for 1 h at 37 °C, followed by washes with Post Ligation Wash Buffer (2,000,420) and 2× saline-sodium citrate buffer at 57 °C.

Sample RNA was then digested with RNase for 30 min at 37 °C, followed by tissue permeabilization and degradation for 40 min at 37 °C. Ligated probes were then captured by the barcoded poly(dT) capture sequences on the slide surface and extended to double-sided DNA for 15 min at 45 °C. Extended probes were then eluted from the slide surface by incubating with 0.08 M KOH for 10 min at room temperature, after which the eluted probes were transferred from the slide to PCR strip tubes and neutralized with 1 M Tris–HCl pH 7.0.

Prior to gene expression library construction, quantitative PCR was performed on the eluted probe DNA to determine the appropriate PCR cycle number needed per sample. Following qPCR, probe DNA from the samples was PCR amplified with Dual Index TS Set A (3,000,511) adapters to make Illumina-compatible libraries for sequencing. Libraries were pooled and sequenced with a target of 25,000 reads/cell using an Illumina NextSeq P2 flow cell.

### scRNA-seq analysis

The 10× Cell Ranger pipeline v7.1.0 was used for FASTQ generation, library QC, gene quantification, and alignment of single cell sequencing data. Downstream analysis of scRNA-seq data was performed using R (v.4.3.3) and the Seurat (v5) package [13, 21]. Data from each sample was filtered to exclude doublets using DoubletFinder [46] and low-quality cells using manual cutoffs for number of reads per cell (< 500 or > 50,000 UMI per cell), number of unique genes per cell (< 500 genes), and percent of reads mapping to mitochondrial probes (> 25%). A 25% cutoff for mitochondrial probes was used because cardiac mitochondrial transcripts comprise ~ 30% of all transcripts due to high energy demands [18, 48]. Data normalization and variance stabilization was performed using SCTransform for each sample [21]. The samples were integrated using the ‘merge’ function in Seurat and batch correction was performed using Harmony [32]. Linear and non-linear dimensionality reduction of integrated data was performed using principal component analysis (PCA) and uniform manifold approximation and projection (UMAP). Clustree [71] was used to determine the optimal resolution for clustering using the Louvain algorithm and a final resolution of 0.1 was chosen. Differentially expressed genes defining each cluster identified using the ‘FindAllMarkers’ function in Seurat, expression of canonical cell type markers, and reference data [2, 35] were used to annotate cell types per cluster. Pairwise differential expression analysis between diabetic and control samples within cell types/clusters was performed using the Wilcoxon Rank Sum test built into the Seurat ‘FindMarkers’ function with a minimum percent expression threshold of 0.1, a minimum average log2-fold difference of 0.25, and Benjamini–Hochberg multiple test corrections [6]. Gene set enrichment analysis (GSEA) and visualization was performed using the clusterProfiler package [69] with the Hallmark and Gene Ontology Biological Processes gene sets from MSigDB [11, 61].

### Visium spatial transcriptomics analysis

The 10X Space Ranger pipeline v 2.0.1 was used for FASTQ generation, library QC, gene quantification, and alignment of Visium sequencing data with the tissue images. Data analysis was performed using R (v.4.3.3) and the Seurat (v5) package [13, 21]. For each sample, data were normalized and the percent of mitochondrial reads was regressed out using SCTransform. Visium data from all samples were integrated using the ‘merge’ function from Seurat and variable features for integration were identified using the ‘SelectIntegrationFeatures’ function. Dimensionality reduction of the integrated data was performed using PCA and UMAP and data were clustered using the Louvain algorithm with a resolution of 0.8. Expression of scRNA-seqcluster/cell type markers was mapped to Visium clusters. Cell types within each spot were deconvoluted using our scRNA-seq data and the Robust Cell Type Decomposition (RCTD) method from the spacexr R package [9]. Spots in regions of interest (ROI) were identified by immunofluorescence staining and their barcodes were identified using the Loupe browser (10X Genomics). ROI included subregions [coronary microvessel (CRM), perivascular (PV), and distal myocardium (MYO) subregions]. We selected MYO spots that were distal (> 100 µm) to observable CRMs. Pairwise differential expression analysis, GSEA, and gene ontology (GO) analysis between ROI of diabetic and control samples were performed as above with a minimum percent expression threshold of 0.25. In plotting differentially expressed genes, gene lists were first sorted by adjusted p-value and then by the absolute value of average log2FC.

### Ligand-receptor interaction analysis

Spearman correlation coefficients of normalized deconvolution weights from spatial transcriptomic data were calculated in diabetic and normal CRM regions of interest to identify cell types that were co-localizing in the same area. Cell–cell pairs with a correlation coefficient of greater than 0.25 were identified. These cell–cell pairs were identified in the scRNA-seq data of normal and diabetic mice and were used for ligand-receptor interaction analysis using the nichenetr R package [7]. Briefly, each cell was considered as a receiver and all cell populations were considered as senders. Only expressed receptors in each receiver cell population and only expressed ligands in sender cell populations were considered for analysis. The gene set of interest for each receiver population was defined as those that are differentially expressed between the diabetic and control conditions for that cell population, assuming that changes in these genes are likely to be influenced by the ligand-receptor interaction. Potential ligand-receptor interactions were prioritized using Differential NicheNet analysis which prioritizes ligands based on six properties: (1) expression of the ligand in a sender cell type compared to all other cell types, (2) expression of the receptor in a receiver cell type, (3) average expression of the ligand gene in the sender, (4) average expression of the receptor gene in the receiver, (5) specificity of the ligand in all cell types in the diabetic condition, and (6) specificity of the receptor in all cell types in the diabetic condition. Final prioritization scores for each ligand-receptor interaction are weighted sums of the scaled prioritization scores for each property described above. Ligands and receptors for indicated cell–cell interactions were used as input for gene set enrichment analysis as described above.

### Coronary resistance microvessel proteomics

Septal CRMs were isolated separately from control heterozygous Db/db and diabetic homozygous db/db mice and were pooled by group (*n* = 4–5 pools of 4 CRMs per biological replicate) for proteomic data collection and analysis. CRMs were lysed by repetitive sonication in 110 µL tissue lysis buffer + protease and phosphatase inhibitors + antifoaming agent. Lysates were kept cold and centrifuged briefly at 4 °C between sonications to move unlysed material to the bottom of the tube. All lysates were centrifuged for 10 min at 4 °C and 14,000 RPM and supernatants were removed to fresh tubes. 16 μg protein of each sample were methanol/chloroform precipitated. Dried samples were resuspended in SDS loading buffer, heated to 70 °C for 5 min, and then separated on a 10% SDS-PAGE gel. The Sypro-stained gel was analyzed by LC–MS/MS (LTQ mass spec followed by 2D LC chromatography – MudPIT; Mascot sequence identification) at the Ohio State University Mass Spec & Proteomics Facility. The number of spectral counts was assessed and compared between normal and diabetic CRMs using a student’s *t* test with a *p* < 0.05 considered to be statistically significant.

## Results

### Single cell landscape of normal and diabetic murine hearts

CMD is a complication of T2D and manifests due to endothelial and smooth muscle dysfunction together with remodeling of the coronary microcirculation. To investigate the cardiovascular transcriptomic changes that occur in the complex cellular landscape of CMD, we isolated hearts from control male heterozygous non-diabetic Db/db mice and male homozygous T2D db/db mice, and we performed paired scRNA-seq and spatial transcriptomic profiling (Graphical Abstract; designed using BioRender).

**Fig. 1:**
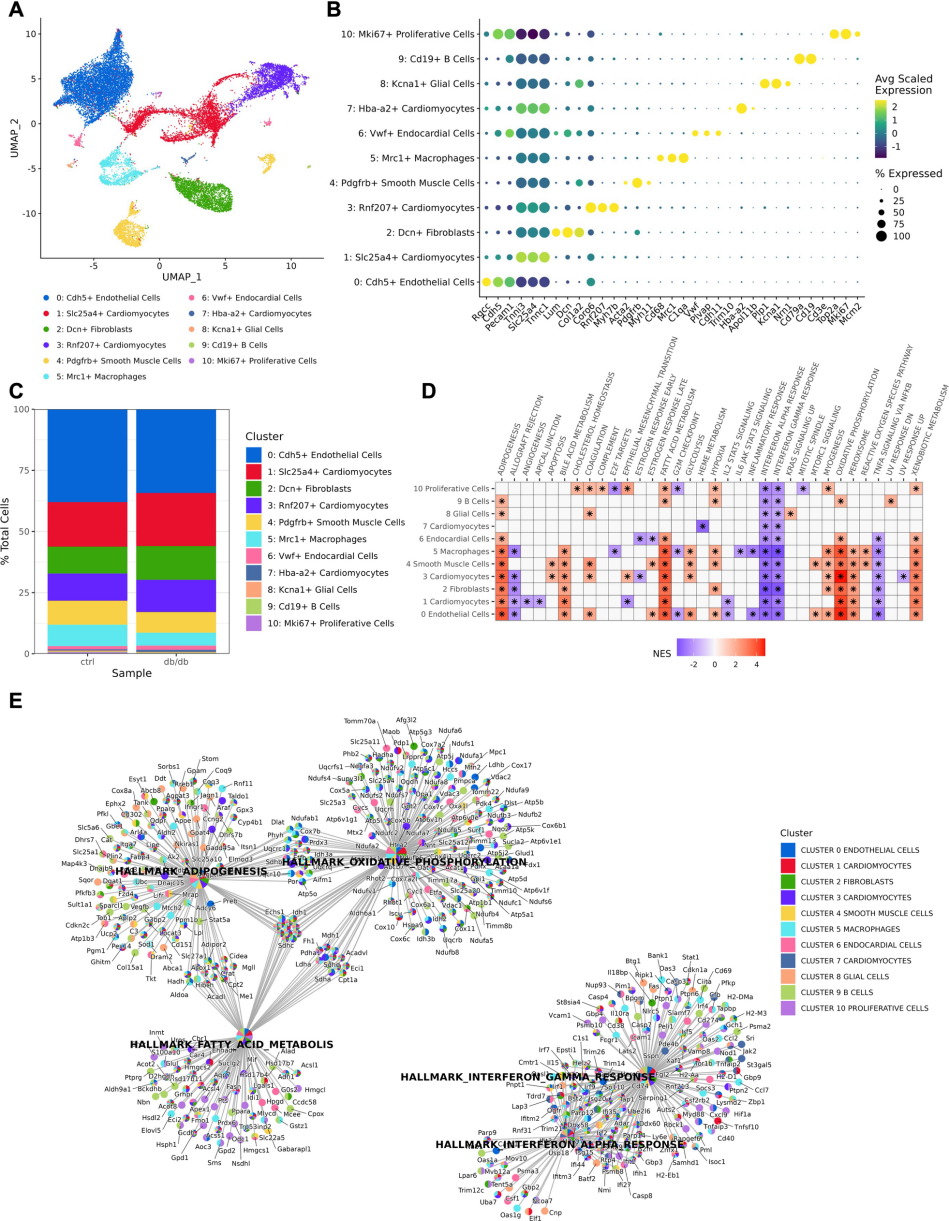
Single-cell transcriptomic profiling of diabetic mouse heart. **A** UMAP plot of cells from diabetic and control heart tissue labeled by cluster number, marker gene, and cell type (*n* = 19,661 cells). **B** Dot plot depicting marker genes for each cluster. **C** Relative proportion of each cluster across samples. **D** Heatmap summary of Hallmark pathways that were enriched in the differentially expressed genes for each cluster between diabetic and control samples. Positive normalized enrichment score (NES) indicates genes associated with the indicated pathway were upregulated in the diabetic samples compared to the control. **E** Gene-concept network plot of the top five most differentially expressed pathways. *n* = 1 per group

In total, 19,661 high-quality single cells were isolated from scrolls of formalin-fixed paraffin-embedded (FFPE) control and diabetic hearts and analyzed by scRNA-sequencing using the 10X Genomics Flex fixed RNA profiling kit (Supplemental Fig. 1). Unsupervised clustering and cell types were assigned using reference data and canonical cell type markers (Supplemental Table 1). We identified 11 distinct cell clusters: endothelial cells, fibroblasts, smooth muscle cells, macrophages, endocardial cells, glial cells, B-cells, Mki67 + proliferative cells, and three clusters of cardiomyocytes (Fig. 1A–B). Cluster 1 was marked by high expression of canonical cardiomyocyte markers *Tnni3*, *Slc25a4*, and *Tnnc1* (cluster 1 *Slc25a4* + cardiomyocytes) (Fig. 1B). Cluster 3 was composed of cardiomyocytes that expressed high levels of *Coro6*, *Rnf207*, *Myh7b* (cluster 3 *Rnf207* + cardiomyocytes) and may represent a cluster of hypertrophic cardiomyocytes (Fig. 1B) [70]. The third cluster of cardiomyocytes expressed *Trim10*, *Hba-a2*, and *Apol11b* (cluster 7 *Hba-a2* + cardiomyocytes) (Fig. 1B). Endothelial cells and *Slc25a4* + cardiomyocytes made up over 50% of the total cells isolated from both the control and diabetic hearts (Fig. 1C).

**Table 1:** Gene set enrichment analysis of scRNA-seq differentially expressed genes. A positive normalized enrichment score (NES) indicates higher expression in the diabetic samples compared to control

Cluster	Pathway	NES	FDR	Core enrichment
0 Endothelial Cells	Fatty acid metabolism	3.788	1.69E−09	Hmgcs2/Acot2/Aoc3/Cd36/Acaa2/Ech1/Decr1/Gpd1/Acadm/Glul/Hsdl2/Me1/Acadl/Aqp7/Car4/Adipor2/Adh1/Eci1/Hadhb/Crat/Idh1/Retsat/Trp53inp2/Suclg1/Acadvl/Mlycd/Etfdh/Aldh9a1/Acox1/Cpt2/Hadh/Acss1/Acads/Acsl1/Mdh1/Eci2/Idh3g/Aco2/Hsd17b4/Sdhc/Mdh2/Echs1/Suclg2/Pdha1/Hsd17b10/Mgll/Fh1/Sdhd/Aldoa/Sdha
1 Cardiomyocytes	Fatty acid metabolism	3.877	1.69E−09	Hmgcs2/Acot2/Hsd17b11/Ehhadh/Decr1/Ech1/Acaa2/Acadm/Cd36/Cidea/Acss1/Gpd1/Hsdl2/Glul/Acadl/Acox1/Aqp7/Eci2/Cpt1a/Eci1/Hadhb/Acadvl/Etfdh/Suclg1/Idh1/Crat/Me1/Acsl1/Retsat/Mlycd/Acads/Hsd17b7/Cpt2/Idh3g/Hsd17b4/Grhpr/Hadh/Mgll/Car4
2 Fibroblasts	Fatty acid metabolism	3.340	1.69E−09	Acot2/Aqp7/Hmgcs2/Ech1/Cd36/Acaa2/Decr1/Car4/Acadm/Hsdl2/Acss1/Acadvl/Acadl/Etfdh/Eci1/Adh1/Cidea/Retsat/Suclg1/Hadhb/Apex1/Mlycd/Eci2/Crat/Gpd1/Acox1/Mgll/Cpt1a/Hsd17b11/Glul/Acsl1/Trp53inp2/Lgals1/Hadh/Acads/Cpt2/Me1/Adipor2/Ccdc58
3 Cardiomyocytes	Fatty acid metabolism	3.867	1.69E−09	Hmgcs2/Acot2/Hsd17b11/Acaa2/Mlycd/Ech1/Decr1/Aqp7/Ehhadh/Acadl/Acadm/Etfdh/Gpd1/Hsdl2/Retsat/Cd36/Lgals1/Cpt2/Cidea/Acads/Crat/Hadhb/Eci1/Acadvl/Suclg1/Eci2/Acss1/Suclg2/Hadh/Trp53inp2/Glul/Mdh1/Fh1/Acsl1/Mdh2/Car4/Cbr1/Sdhc/Echs1/Idh3g/Apex1/Hsd17b4/Mif/Hsd17b10/Aco2/Ldha/Pdha1/Sdha/Adh1/Grhpr/Acox1/Sdhd
4 Smooth Muscle Cells	Fatty acid metabolism	3.756	1.69E−09	Hmgcs2/Acot2/Car4/Aqp7/Cd36/Ech1/Decr1/Acaa2/Fmo1/Glul/Lgals1/Retsat/Acadm/Etfdh/Slc22a5/Cidea/Cpt1a/Me1/Acadl/Hsd17b11/Acss1/Adipor2/Mgll/Idh1/Hadhb/Mlycd/Aldh9a1/Acadvl/Eci1/Hsdl2/Gpd1/Acsl1/Eci2/Acox1/Crat/Suclg1/Hadh/Idh3g/Acads/Hmgcs1/Cpt2
5 Macrophages	Fatty acid metabolism	3.768	1.69E−09	Hmgcs2/Acot2/Car4/Aqp7/Cd36/Decr1/Adh1/Acaa2/Ech1/Cidea/Lgals1/Gpd1/Acadm/Hsdl2/Eci1/Acadl/Acadvl/Idh1/Eci2/Me1/Hadhb/Suclg1/Glul/Mgll/Crat/Acss1/Cpt1a/Acox1/Cpt2/Etfdh/Gabarapl1/Acsl1/Mlycd/Fmo1/Hadh/Acads/Mdh1/Hibch/Aldh9a1/Echs1/Suclg2/Sms/Mdh2/Aco2/Idh3g/Retsat/S100a10/Gstz1/Elovl5/Prdx6/Hsd17b4/Uros/Grhpr/Trp53inp2/Adipor2
6 Endocardial Cells	Fatty acid metabolism	2.357	1.70E−05	Hmgcs2/Cidea/Apex1/Acot2/Car4/Adh1/Decr1/Hpgd/Fmo1/Ech1/Cd36/Gpd1/Mcee/Etfdh/Retsat/G0s2/Hmgcs1/Acadm/Acss1/Hadhb/Acaa2/Uros/Eci1/Acadl/Eci2/Glul/Hsd17b4/Hsdl2/Hsd17b11
0 Endothelial Cells	Oxidative phosphorylation	4.582	1.69E−09	Pdk4/Slc25a20/Acaa2/Ech1/Decr1/Atp5a1/Acadm/Hadha/Iscu/Eci1/Hadhb/Idh1/Retsat/Suclg1/Cox6c/Acadvl/Etfdh/Idh2/Cox4i1/Etfb/Aldh6a1/Ndufc2/Mgst3/Nnt/Bckdha/Ndufa3/Atp5g3/Ndufa9/Etfa/Mdh1/Slc25a4/Ndufs6/Idh3g/Aco2/Idh3a/Acat1/Uqcrfs1/Ogdh/Oat/Ndufs1/Ndufa6/Uqcrq/Slc25a3/Cox5b/Sdhc/Timm10/Ndufa7/Got2/Ndufb8/Atp5d/Mdh2/Phyh/Cox7a2/Ndufa8/Sdhb/Atp5b/Ndufb2/Uqcrc2/Ndufc1/Vdac1/Fdx1/Echs1/Cox7c/Ndufb3/Cox5a/Acadsb/Slc25a11/Ndufab1/Ndufv1/Pdha1/Uqcrb/Cox17/Hsd17b10/Atp1b1/Fh1/Ndufs7/Mfn2/Ndufv2/Sdhd/Prdx3/Uqcr10/Atp5o/Sdha/Uqcrc1/Dlat/Ndufa1/Atp5l/Cox10/Ldhb/Atp5c1/Hspa9/Phb2/Ndufs4/Idh3b/Ndufa2/Slc25a12/Atp5j2/Mtx2/Cpt1a/Ndufb4/Ndufs2/Cox7b
1 Cardiomyocytes	Oxidative phosphorylation	4.198	1.69E−09	Pdk4/Decr1/Ech1/Slc25a20/Acaa2/Acadm/Hadha/Maob/Atp5a1/Cpt1a/Eci1/Hadhb/Acadvl/Etfdh/Suclg1/Idh1/Phb2/Retsat/Cox6c/Idh2/Bckdha/Ndufa9/Idh3g/Nnt/Mgst3/Ndufa7/Acat1/Iscu/Cox4i1/Cox17/Etfa/Fh1/Ndufa3/Aco2/Etfb/Ndufa6/Ndufc2/Timm10/Uqcrq/Cox10/Aldh6a1/Mdh2/Surf1/Oat/Ndufs6/Mdh1/Atp5d/Uqcrfs1
2 Fibroblasts	Oxidative Phosphorylation	3.587	1.69E−09	Pdk4/Ech1/Slc25a20/Acaa2/Decr1/Atp5a1/Acadm/Hadha/Acadvl/Etfdh/Eci1/Cox6c/Retsat/Suclg1/Hadhb/Ndufb3/Cpt1a/Timm10/Ndufa3/Mgst3/Idh2/Afg3l2/Cox4i1/Acat1/Ndufa9/Uqcrq/Ndufs6/Supv3l1/Idh3g/Aco2/Ndufa7/Ndufa6/Nnt/Atp5g3/Mtx2/Etfb/Bckdha/Vdac1/Mdh1/Cox17/Idh1/Etfa/Uqcrb/Ndufs4/Aifm1/Got2/Ndufc1/Atp5c1/Sdhc/Cox7b/Aldh6a1/Slc25a3/Idh3a/Slc25a4/Pdhx/Atp5l/Cox11/Slc25a12/Cox5b/Atp5d/Ndufc2/Opa1/Sdhd/Ogdh/Phb2/Ndufb8/Atp5b/Atp5o/Slc25a11
3 Cardiomyocytes	Oxidative phosphorylation	4.738	1.69E−09	Pdk4/Slc25a20/Acaa2/Maob/Ech1/Atp5a1/Decr1/Hadha/Mgst3/Acadm/Etfdh/Iscu/Retsat/Hadhb/Eci1/Acadvl/Suclg1/Oat/Cox4i1/Ndufa3/Etfb/Ndufa7/Atp5g3/Ndufa9/Cox6c/Ndufs6/Mdh1/Cox5b/Ndufb8/Cox7a2/Ndufc2/Bckdha/Fh1/Vdac1/Mdh2/Uqcrfs1/Idh2/Sdhc/Got2/Cox7c/Echs1/Idh3g/Uqcrq/Atp5b/Ndufc1/Atp5o/Uqcrc2/Acat1/Slc25a3/Atp5d/Hsd17b10/Cox5a/Cox17/Prdx3/Aldh6a1/Aco2/Ldha/Pdha1/Pdhx/Sdha/Ndufa8/Slc25a4/Sdhd/Hspa9/Uqcrc1/Vdac3/Ndufa6/Ndufb7/Ndufb2/Atp5l/Etfa/Atp5c1/Timm10/Atp6v1c1/Idh3a/Ogdh/Ndufb3/Fdx1/Htra2/Ndufb6/Phb2/Nnt/Slc25a11/Ndufa1/Atp1b1/Ndufa2/Sucla2/Opa1/Ndufs7/Por/Uqcrb/Acaa1a/Gpi1/Cox6b1/Mtx2/Ndufs2
4 Smooth Muscle Cells	Oxidative phosphorylation	3.358	1.69E−09	Pdk4/Slc25a20/Ech1/Decr1/Acaa2/Atp5a1/Retsat/Iscu/Acadm/Etfdh/Cpt1a/Hadha/Idh1/Hadhb/Acadvl/Eci1/Cox6c/Mgst3/Suclg1/Abcb7/Nnt/Idh3g/Idh2/Ndufa7/Bckdha/Vdac1/Por/Atp1b1/Idh3a/Cox17/Ndufa3/Cox4i1/Etfb/Uqcrq/Atp6v0e/Ndufc2/Ndufs6/Ndufb3/Oat/Ndufa9/Aco2/Uqcrb/Etfa/Mdh2/Ogdh/Slc25a4/Slc25a3/Acat1/Ndufc1/Surf1/Ndufa6/Sdhd/Atp5g3
5 Macrophages	Oxidative phosphorylation	3.971	1.69E−09	Pdk4/Slc25a20/Decr1/Acaa2/Atp5a1/Ech1/Acadm/Eci1/Acadvl/Idh1/Hadha/Hadhb/Suclg1/Aldh6a1/Iscu/Cpt1a/Etfdh/Cox6c/Idh2/Atp6v1f/Ndufa9/Mgst3/Cox17/Etfa/Cox4i1/Mdh1/Etfb/Uqcrq/Nnt/Ndufa7/Echs1/Bckdha/Atp5g3/Cox5b/Mdh2/Aco2/Idh3g/Retsat/Ndufa3/Ndufs1/Uqcrfs1/Atp5d/Hspa9/Timm13/Ndufs6/Acat1/Ndufa6/Oat/Vdac1/Sdhc/Sucla2/Ndufv2/Ogdh/Ldha/Uqcrc2/Atp5l/Ndufv1/Uqcr10/Slc25a3/Slc25a4/Mtx2/Idh3a/Aifm1/Atp5b/Sdhb/Dlst/Cox5a/Got2/Uqcrc1/Hsd17b10/Ndufc2/Phb2/Atp5o/Ndufs2/Ndufab1/Ndufb8/Slc25a12/Vdac3/Phyh/Cox7b/Lrpprc/Rhot2/Dld/Dlat/Pdhx/Ndufs7/Hccs
6 Endocardial Cells	Oxidative phosphorylation	2.035	3.9e−04	Pdk4/Bckdha/Decr1/Slc25a20/Ech1/Pdp1/Etfdh/Atp5a1/Retsat/Mgst3/Rhot2/Tomm70a/Acadm/Hadhb/Acaa2/Eci1/Vdac3/Ndufc2/Uqcrq/Hadha/Opa1/Nnt/Uqcrfs1/Cox7a2l/Nqo2/Mpc1/Acadvl/Ndufb3/Pdhx/Iscu/Ndufa6/Ndufb2/Cox5b/Cox6c/Cox4i1/Aco2/Oxa1l/Atp6v1g1/Atp5d/Cox11/Idh3g/Mfn2/Cycs/Ndufs7/Mdh1/Sdhb/Ndufb5/Ldha/Etfb/Ndufa3/Got2/Etfa/Rhot1/Cyc1/Ndufb6/Fh1/Ndufv2/Vdac2/Slc25a3/Suclg1/Ndufs4/Tomm22/Acat1/Ndufa2/Oat
0 Endothelial Cells	Adipogenesis	3.876	1.69E−09	Angptl4/Ephx2/Fabp4/Cd36/Pparg/Acaa2/Ucp2/Ech1/Decr1/Slc27a1/Acadm/Pim3/Me1/Plin2/Acadl/Cat/Adipor2/Stom/Crat/Idh1/Retsat/Cyp4b1/Lpl/Suclg1/Lpcat3/Acox1/Gpam/Cpt2/Hadh/Acads/Arl4a/Etfb/Sod1/Mgst3/Bckdha/Agpat3/Gpx3/Itga7/Fzd4/Idh3g/Aco2/Idh3a/Coq3/Sult1a1/Vegfb/Uqcrq/Tob1/Sdhc/Mdh2/Phyh/Lipe/Sdhb/Pgm1/Sorbs1/Echs1/Preb/Ghitm/Ndufab1/Pfkl/Mgll/Prdx3/Uqcr10/Atp5o/Aldoa/Uqcrc1/Dlat
1 Cardiomyocytes	Adipogenesis	3.040	1.74E−09	Angptl4/Ephx2/Decr1/Ech1/Slc27a1/Cat/Acaa2/Elmod3/Lpcat3/Acadm/Cd36/Cidea/Lipe/Fabp4/Ucp2/Gpam/Pparg/Agpat3/Acadl/Acox1/Dgat1/Pim3/Suclg1/Idh1/Crat/Me1/Retsat/Bckdha/Acads/Pfkl/Cpt2/Idh3g/Mgst3/Hadh/Mgll/Cyp4b1/Stom/Plin2/Sod1/Dnajb9/Ghitm/Fzd4/Aco2/Etfb
2 Fibroblasts	Adipogenesis	3.143	1.69E−09	Angptl4/Ephx2/Fabp4/Pparg/Cyp4b1/Ech1/Plin2/Cd36/Acaa2/Decr1/Slc27a1/Sult1a1/Cat/Ucp2/Acadm/Acadl/Lpcat3/Lipe/Cidea/Retsat/Agpat3/Suclg1/Abcb8/Crat/Acox1/Pim3/Mgll/Pfkl/Mgst3/Gpam/Jagn1/Stom/Hadh/Acads/Cpt2/Me1/Pfkfb3/Sod1/Adipor2/Uqcrq/Idh3g/Aco2/Dgat1/Cd302/Itga7
3 Cardiomyocytes	Adipogenesis	3.587	1.69E−09	Angptl4/Cyp4b1/Lipe/Acaa2/Ephx2/Cat/Ech1/Lpcat3/Decr1/Sult1a1/Fabp4/Agpat3/Gadd45a/Mgst3/Acadl/Acadm/Ucp2/Stom/Retsat/Cd36/Cpt2/Cidea/Acads/Crat/Slc27a1/Suclg1/Sod1/Gpam/Etfb/Plin2/Hadh/Dhrs7b/Arl4a/Pim3/Ghitm/Bckdha/Vegfb/Mdh2/Taldo1/Sdhc/Echs1/Idh3g/Uqcrq/Atp5o/Prdx3/Aco2/Elmod3/Gpx3/Cd151/Acox1/Pparg/Uqcrc1/Ndufb7/Fzd4/Dnajb9/Qdpr/Aldh2/Lpl/Mgll/Dnajc15/Ifngr1/Idh3a/Pfkl
4 Smooth Muscle Cells	Adipogenesis	3.469	1.69E−09	Angptl4/Plin2/Ephx2/Fabp4/Lipe/Cd36/Ech1/Pparg/Decr1/Acaa2/Ucp2/Cat/Pim3/Retsat/Slc27a1/Mrap/Acadm/Cidea/Sult1a1/Me1/Acadl/Lpcat3/Gpx3/Adipor2/Tkt/Mgll/Idh1/Fzd4/Gpam/Cd151/Sod1/Mgst3/Acox1/Crat/Esyt1/Suclg1/Coq3/Hadh/Idh3g/Acads/Cd302/Cpt2/Bckdha/Por/Lpl/Sparcl1/Idh3a/Ifngr1/Vegfb/Etfb/Uqcrq/C3
5 Macrophages	Adipogenesis	3.264	1.69E−09	Angptl4/Ephx2/Gpx3/Slc27a1/Fabp4/Cd36/Decr1/Acaa2/Cyp4b1/Ech1/Cidea/Plin2/Lpcat3/Pim3/Acadm/Acadl/Idh1/Me1/Gpam/Suclg1/Mgll/Crat/Acox1/Mtch2/Ucp2/Cpt2/Cat/Mgst3/Hadh/Acads/Sod1/Cmbl/Hibch/Lipe/Fzd4/Etfb/Uqcrq/Agpat3/Lpl/Echs1/Bckdha/Sult1a1/Stom/Mdh2/Elmod3/Aco2/Idh3g/Retsat/Vegfb/Pgm1/Apoe/Adipor2/Cd151/Dram2/Aldoa/Sdhc/Sorbs1/Lifr/Uqcr10/Gbe1/Idh3a/Aifm1/Itga7/Sdhb/Qdpr/Rnf11/Ubc/Uqcrc1/Ppm1b/Adcy6/Atp5o/Ndufab1/Coq9/Phyh/Ghitm/Cox7b/Pfkl/Dnajb9/Aldh2/Dld/Dlat/Phldb1
6 Endocardial Cells	Adipogenesis	1.890	5.10e−04	Angptl4/Cidea/Pparg/Ephx2/Mrap/Bckdha/Decr1/Lipe/Ccng2/Gpam/Ech1/Fabp4/Cd36/Sult1a1/Retsat/Mgst3/Acadm/Tkt/Pfkfb3/Acaa2/Esyt1/Atp1b3/Lifr/Acadl/Pqlc3/Dhrs7/Fzd4
0 Endothelial Cells	Interferon Alpha Response	− 3.072	1.69E−09	Casp1/Ifitm2/Il15/Parp9/Parp12/Ogfr/Rnf31/Pnpt1/Ube2l6/Nmi/Ifi35/Ifitm3/Eif2ak2/Trim14/Stat2/Lgals3bp/Cmpk2/Ifih1/Gbp3/Samd9l/Ddx60/Parp14/Ripk2/Herc6/Dhx58/Bst2/Rtp4/Cd74/B2m/Ifit3/Irf7/Ifit2/Usp18/Isg15/Oas1a/Ifi44/H2-Q7
1 Cardiomyocytes	Interferon Alpha Response	− 3.091	1.69E−09	Psmb8/Ifitm2/Helz2/Elf1/Ly6e/Mvb12a/Psmb9/Stat2/Lgals3bp/Tdrd7/Trim26/Uba7/Parp12/Parp9/Gbp2/Rsad2/Ifitm3/Irf9/Parp14/Ifih1/Gbp3/Bst2/Rtp4/Irf7/B2m/Cd74/Ifit3/Isg15
2 Fibroblasts	Interferon Alpha Response	− 3.577	1.69E−09	Parp12/Ifih1/Adar/Mov10/Parp9/Ube2l6/Cmtr1/Psmb9/Helz2/Trim21/Isg20/Eif2ak2/Tdrd7/Trim14/Psmb8/Trim12c/Ifi35/Ddx60/Ly6e/Parp14/Irf9/Rsad2/Cmpk2/Lgals3bp/Stat2/Gbp2/Ifit2/Herc6/Gbp3/B2m/Samd9l/Irf7/Bst2/Ifit3/Cd74/Rtp4/Isg15
3 Cardiomyocytes	Interferon Alpha Response	− 2.966	1.69E−09	Psmb8/Trim26/Psmb9/Gbp2/Tap1/Ifi35/Ly6e/Helz2/Lgals3bp/Stat2/Rsad2/Samd9l/Il15/Bst2/Ifitm3/Ifi30/Uba7/Parp14/Irf9/Nmi/Ifih1/Parp9/Gbp3/Cd74/Ifit3/Rtp4/B2m/Herc6/Irf7/Ddx60/H2-Q7/Isg15
4 Smooth Muscle Cells	Interferon Alpha Response	− 2.880	6.66E−08	Lgals3bp/Parp12/Pnpt1/Ube2l6/Cmtr1/Irf2/Helz2/Psmb8/Ly6e/Ifih1/Bst2/Parp9/Herc6/Adar/Eif2ak2/Ifi27/Parp14/Gbp3/Irf9/Sp110/Stat2/Cd74/Rtp4/Irf7
5 Macrophages	Interferon Alpha Response	− 3.486	1.69E−09	Sp110/Trim26/Mvb12a/Ogfr/Il15/Trim21/Uba7/Gbp2/Gbp3/Ifitm3/Rnf31/Ube2l6/Eif2ak2/Trim14/Epsti1/Parp12/Ly6e/Ifi35/Psmb9/Irf9/Helz2/Tap1/Samd9l/Parp9/Adar/Psmb8/Ifih1/B2m/H2-Q7/Bst2/Rsad2/Stat2/Cmpk2/Parp14/Cd74/Ddx60/Mov10/Lgals3bp/Herc6/Oas1a/Dhx58/Usp18/Rtp4/Oasl1/Isg15/Ifit3/Oas1g/Irf7
6 Endocardial Cells	Interferon Alpha Response	− 2.278	2.18E−05	Psma3/Csf1/Trim26/Mvb12a/Lap3/Pnpt1/Ddx60/Ube2l6/Ly6e/Lgals3bp/B2m/Samd9l/Herc6/Irf7/Casp8/Gbp3/Bst2/C1s1/Ifit3/Parp9/Ifit2/Rtp4/Ifi44/Cd74/H2-Q7/Isg15
0 Endothelial Cells	Interferon Gamma Response	− 2.972	1.69E−09	H2-M3/Lysmd2/Rnf31/Pnpt1/Ube2l6/Tnfaip3/Nmi/Icam1/Ifi35/Ifitm3/Eif2ak2/Trim14/Stat2/Pml/Lgals3bp/Gch1/Cmpk2/Ifih1/Gbp3/H2-D1/Samd9l/Casp4/Ddx60/Parp14/Ripk2/Herc6/Pde4b/Dhx58/Xaf1/Cxcl9/Bst2/Rtp4/Cd74/B2m/Rnf213/Ifit3/Irf7/Ifit2/Usp18/Isg15/Ifi44/H2-Q7/Oas2
1 Cardiomyocytes	Interferon Gamma Response	− 2.678	4.56E−09	Samhd1/Stat1/Rbck1/Psmb8/Auts2/Ifitm2/Tnfsf10/Helz2/Lysmd2/Ly6e/Psmb9/St8sia4/Stat2/Lgals3bp/Cd38/Tdrd7/Trim26/Fgl2/Parp12/H2-D1/Gbp9/Rsad2/Ifitm3/Irf9/Parp14/Ifih1/Gbp3/Bst2/Rtp4/Rnf213/Irf7/B2m/Xaf1/H2-Eb1/Cd74/Ifit3/H2-Aa/Isg15
2 Fibroblasts	Interferon Gamma Response	− 3.468	1.69E−09	Fgl2/Samhd1/Parp12/Ifih1/Adar/Tapbp/Ube2l6/Cmtr1/Pml/Psmb9/H2-M3/Il18bp/Psmb10/Helz2/Tnfaip2/Stat1/Trim21/Isg20/Eif2ak2/Pde4b/Tdrd7/Trim14/Psmb8/Ifi35/Ddx60/Ly6e/Parp14/Irf9/Rsad2/Cmpk2/Lgals3bp/Stat2/Ifit2/Herc6/Gbp3/H2-D1/Rnf213/B2m/Samd9l/Irf7/Xaf1/Bst2/Ifit3/Cd74/Rtp4/Isg15
3 Cardiomyocytes	Interferon Gamma Response	− 2.606	3.16E−08	Ripk1/Psmb8/Trim26/Pim1/Psmb9/Samhd1/St8sia4/Tap1/Ifi35/Ly6e/Helz2/Psmb10/Lgals3bp/Ptpn2/Stat2/Rsad2/Nlrc5/Samd9l/Il15/Bst2/Ifitm3/Ifi30/Rnf213/Parp14/Irf9/Xaf1/H2-D1/Nmi/Fgl2/H2-Aa/Ifih1/Gbp3/Cd74/Ifit3/Rtp4/B2m/H2-Eb1/Herc6/Irf7/Ddx60/H2-Q7/Isg15/Oas2
4 Smooth Muscle Cells	Interferon Gamma Response	− 2.692	2.82E−07	Samhd1/Lgals3bp/Parp12/Pnpt1/Ube2l6/Cmtr1/Irf2/Psmb10/Pml/Helz2/Psmb8/Ly6e/Pde4b/Stat1/Ifih1/Bst2/Herc6/Adar/Eif2ak2/Ifi27/Parp14/Xaf1/Gbp3/Irf9/Sp110/Stat2/Rnf213/Cd74/Rtp4/Irf7
5 Macrophages	Interferon Gamma Response	− 3.592	1.69E−09	Ifitm3/Rnf31/Ube2l6/Tor1b/Ptpn6/Gch1/Isoc1/Eif2ak2/Trim14/Auts2/Epsti1/Pde4b/Bank1/Parp12/Icam1/Ly6e/Fgl2/H2-M3/Ifi35/Il10ra/Psmb9/Nlrc5/Irf9/Cd40/Helz2/Pml/H2-D1/Tap1/Samd9l/Adar/Stat1/Psmb8/Ifih1/Csf2rb2/B2m/H2-Q7/Fcgr1/Slamf7/Znfx1/Bst2/Ccl2/Rsad2/Stat2/Cmpk2/Parp14/Cd74/Ddx60/Ciita/Casp3/H2-Eb1/Lgals3bp/H2-DMa/Herc6/H2-Aa/Dhx58/Usp18/Rnf213/Xaf1/Rtp4/Cfb/Oasl1/Isg15/Ccl7/Ifit3/Oas2/Zbp1/Irf7
6 Endocardial Cells	Interferon Gamma Response	− 1.930	4.13E−04	Rnf213/Casp4/Lap3/Pnpt1/Ddx60/Lats2/H2-D1/Ube2l6/Xaf1/Ly6e/Lgals3bp/Serping1/St8sia4/B2m/Ripk1/Samd9l/Cfb/Irf5/Herc6/Irf7/Casp8/Nup93/Gbp3/Bst2/C1s1/Ifit3/Ifit2/Socs3/H2-Eb1/Rtp4/Ifi44/Cd74/H2-Aa/H2-Q7/Isg15/Oas2

Please reference Supplemental Table 3 for full dataset. False discovery rate (FDR) represents *p* value adjustment

To identify transcriptomic changes in each cell type, we performed differential expression analysis comparing diabetic and control samples (Supplemental Table 2). Gene set enrichment analysis (GSEA) was used to identify changes in the Hallmark gene signatures (Table 1; Supplemental Table 3). In all cell clusters, there was a significant downregulation of genes associated with interferon alpha and gamma signaling, including master transcriptional regulators *Stat1/2* and *Jak2,* in the diabetic sample compared to the control (Fig. 1D–E). Genes related to adipogenesis, fatty acid metabolism, and oxidative phosphorylation were upregulated in cluster 0 *Cdh5* + endothelial cells, cluster 1 *Slc25a4* + cardiomyocytes, cluster 2 *Dcn* + fibroblasts, cluster 3 *Rnf207* + cardiomyocytes, cluster 4 *Pdgfrb* + smooth muscle cells, cluster 5 *Mrc1* + macrophages, cluster 6 *Vwf* + endocardial cells, and cluster 9 *Cd19* + B-cells (Fig. 1D).

**Table 2:** Visium CRM gene set enrichment analysis

Description	NES	*p* Adjusted	Core enrichment
Interferon Alpha Response	− 2.596	2.09e−05	Ifitm2/Psma3/Tmem140/Ifi27/Ifitm3/Cmtr1/Parp12/Adar/Helz2/Irf2/B2m/Bst2/Gbp3/Lgals3bp/Cd74
Fatty Acid Metabolism	2.563	6.80e−05	Hmgcs2/Acot2/Adh1/Aqp7/Ech1/Decr1/Mlycd/Hsdl2/Cpt1a/Acadvl/Car4/Acaa2/Acss1/Eci1/Cidea/Acadl/Hsd17b11/Hadh/Alad/Eci2/Retsat/Acsl1/Cd36/Acadm/Acox1/Mgll
Oxidative Phosphorylation	2.380	7.16e−04	Pdk4/Slc25a20/Ech1/Maob/Decr1/Cpt1a/Acadvl/Acaa2/Hadha/Eci1/Timm17a/Atp6v1g1/Retsat/Acadm/Fdx1/Aldh6a1/Lrpprc/Acaa1a/Nqo2/Idh1/Idh2/Sdhc/Phb2/Nnt/Timm8b/Rhot1/Ndufs1/Atp5a1/Ndufs3/Etfdh/Sdhd/Oat/Prdx3/Afg3l2/Uqcrc1/Bax/Idh3a
Adipogenesis	2.145	9.79e−04	Ucp2/Cyp4b1/Dhrs7b/Lipe/Ech1/Sult1a1/Decr1/Slc27a1/Acaa2/Cat/Pim3/Cidea/Ephx2/Acadl/Pfkl/Hadh/Lpcat3/Retsat/Stom/Cd36/Elmod3/Acadm/Adcy6/Acox1/Mgll/Fabp4/Gpam/Acads/Agpat3/Idh1/Lifr/Aldh2/Sdhc
Interferon Gamma Response	− 2.146	2.94e−03	Cfh/Ifitm2/Psma3/Xaf1/Cmklr1/Ifi27/Ifitm3/Nfkb1/Cmtr1/Fgl2/Parp12/Adar/Helz2/Irf2/B2m/Bst2/Rnf213/Gbp3/Lgals3bp/Cd74
Peroxisome	1.930	2.57e−02	Ech1/Mlycd/Slc35b2/Cat/Ephx2/Hsd17b11/Eci2/Retsat/Acsl1/Ercc1/Acox1/Pex13/Smarcc1/Acaa1a/Idh1/Idh2
Xenobiotic Metabolism	1.853	3.15e−02	Pdk4/Adh1/Serpine1/Nqo1/Ptgds/Ech1/Cat/Aox1/Entpd5/Slc6a6/Retsat/Lonp1/Dhps/Cd36/Sar1b/Ninj1/Csad/Comt/Acox1
Kras Signaling DN	1.954	4.00e−02	Myh7/Sgk1/Zbtb16/Tcf7l1/Fgf16/Ybx2
Bile Acid Metabolism	1.882	4.00e−02	Lipe/Mlycd/Slc35b2/Cat/Ephx2/Hsd17b11/Pex16/Retsat/Acsl1/Pex13/Optn/Pex7

A positive normalized enrichment score (NES) indicates higher expression in the diabetic sample compared to control. A negative NES indicates lower expression in the diabetic sample compared to control. Please reference Supplemental Table 5 for full dataset

**Table 3:** Proteomics validation dataset (selected from full set)

Gene/Protein	Spatial transcriptomics				Proteomics		
	Control expression	db/db expression	avg_log2 FC	*p* adjusted	Control spectral count	db/db spectral count	*p* value
Fabp3	1	1	0.598	3.70e−23	ND	ND	
Ech1	1	1	0.897	2.61e−19	1	4	1.70e−02
Myh7	0.583	0.898	2.722	2.45e−15	323	328	8.90e−01
Acot2	0.725	0.918	1.771	2.67e−14	0	1	2.70e−01
Acaa2	0.992	1	0.742	4.58e−14	6	17	3.60e−03
Decr1	1	1	0.820	1.38e−13	2	9	1.30e−02
Slc25a20	0.825	0.98	1.249	7.88e−13	0	2	1.60e−03
Acadl	1	1	0.649	3.53e−12	7	12	2.40e−02
Myom2	1	1	0.783	5.06e−12	ND	ND	
B2m	1	0.898	− 1.056	8.61e−12	ND	ND	
Cd74	0.817	0.337	− 2.381	1.18e−11	ND	ND	
Eci1	1	0.99	0.720	7.58e−11	5	12	2.40e−02
Mt1	0.983	1	0.912	9.32e−11	ND	ND	
Pdk4	0.967	0.98	1.676	2.17e−09	ND	ND	
Eno3	1	1	− 0.679	3.28e−09	ND	ND	
Cox7a1	1	1	− 0.414	5.63e−09	5	2	1.30e−03
Cd36	1	1	0.492	1.23e−08	2	3	3.00e−01
Hadha	0.983	1	0.731	1.42e−08	60	52	4.40e−01
Dbi	0.942	1	0.863	2.13e−08	ND	ND	
Ptgds	0.842	0.98	0.924	5.21e−08	ND	ND	
Acadvl	0.967	1	0.760	2.58e−07	12	24	2.00e−02
Psap	1	1	0.516	2.78e−07	ND	ND	
Cox6a2	1	1	0.311	4.27e−07	ND	ND	
Acadm	1	1	0.490	5.64e−07	1	8	2.40e−03
Myh6	1	1	− 0.277	1.29e−06	ND	ND	
Acot1	0.092	0.48	2.615	1.34e−06	ND	ND	

*ND* not detected in proteomics screen

### Spatial mapping of cell types in the normal and diabetic heart

To investigate the spatial landscape of these changes in gene expression in CRMs, perivascular, and myocardial regions, we performed spatial transcriptomic analysis of heart cross-sections from control and diabetic mice using the 10X Genomics Visium Spatial Gene Expression FFPE kit. Tissue from each sample covered an average of 1,618 spots (Supplemental Fig. 2). We used the corresponding scRNA-seq data, described above, to map annotated cell clusters to the tissue using Robust Cell Type Decomposition (RCTD). Nearly all spots contained cells identified as cluster 1 *Slc25a4* + cardiomyocytes in control and diabetic hearts (Supplemental Fig. 3A–B). Cluster 6 *Vwf* + endocardial cells were predominantly found in the endocardium (Supplemental Fig. 3A–B). Cluster 2 *Dcn* + fibroblasts and cluster 4 *Pdgfrb* + smooth muscle cells were largely located surrounding the CRMs in the perivascular (PV) space (Supplemental Fig. 3A–B). In contrast, cluster 3 *Rnf207* + cardiomyocytes appeared to be largely excluded from the area surrounding CRMs (Supplemental Fig. 3A–B), particularly in control samples. Interestingly, when looking at the heart as a whole, diabetic samples had a significantly higher deconvolution weight for the cluster 3 *Rnf207* + cardiomyocytes compared to control samples (Supplemental Fig. 3C).

**Fig. 2:**
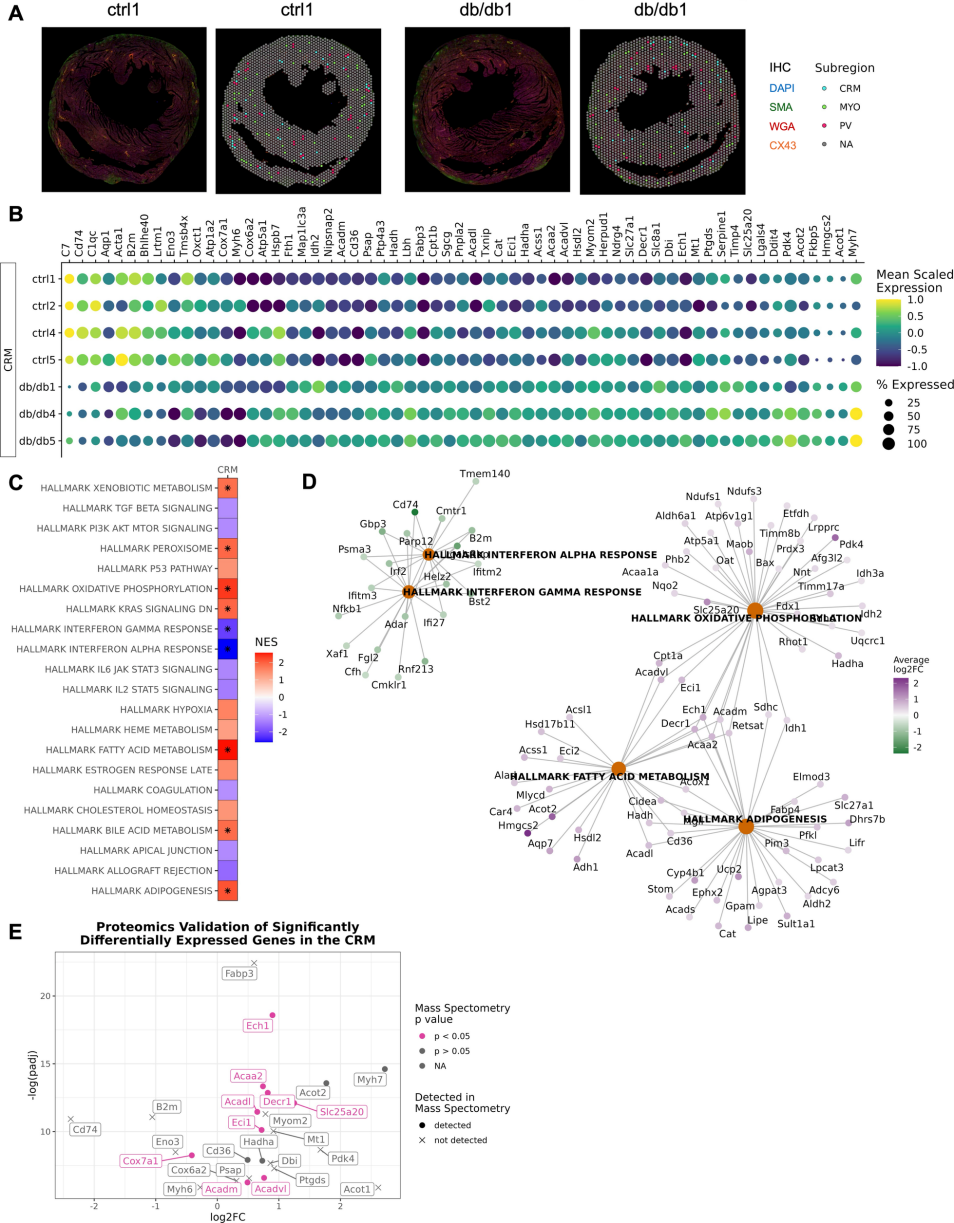
Identification of transcriptomic differences in coronary resistance microvessels (CRM) in normal and diabetic hearts. **A** Immunohistochemical staining of nuclei (DAPI), cell membranes (WGA), smooth muscle cells (SMA), and gap junctions (CX43) guided the selection of coronary resistance microvessels (CRM), perivascular regions (PV), and myocardium (MYO) subregions. **B** Dot plot depicting significantly differentially expressed genes in CRM subregions of the diabetic and normal hearts. **C** Heatmap of Hallmark pathways enriched in differentially expressed genes. **D** Gene concept map of the top five most differentially expressed pathways in the CRM subregions. *n* = 3–4 per group. **E** Volcano plot of genes that were significantly differentially expressed in CRM subregions of the diabetic and normal hearts with validated differentially expressed proteins highlighted

**Fig. 3:**
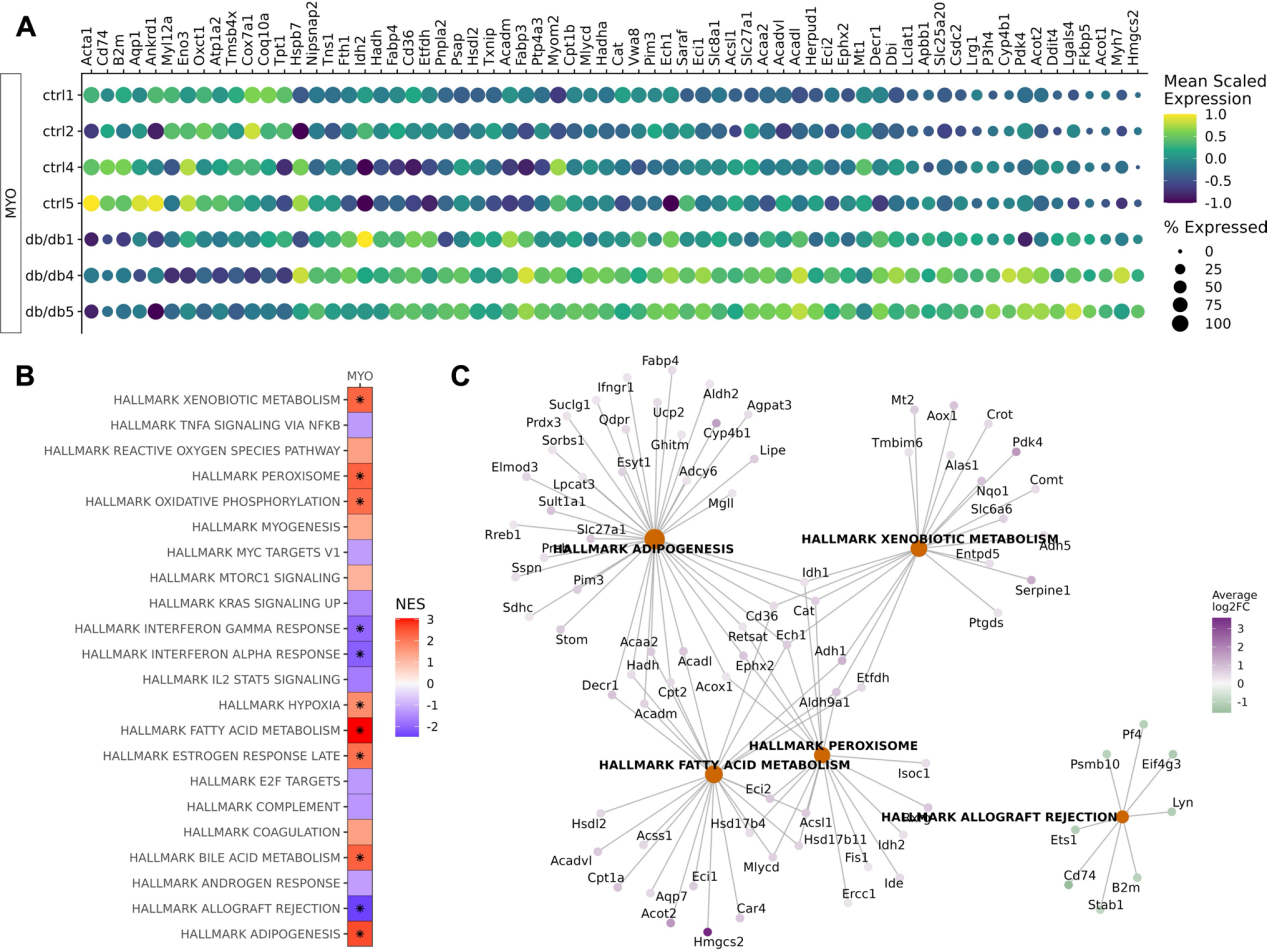
Identification of transcriptomic differences in myocardium (MYO) in normal and diabetic hearts. **A** Dot plot depicting significantly differentially expressed genes in MYO subregions of the diabetic and normal hearts. **B** Heatmap of Hallmark pathways enriched in differentially expressed genes. **C** Gene concept map of the top five most differentially expressed pathways in the MYO subregions. *n* = 3–4 per group

### Gene expression changes in diabetic heart

We performed immunohistochemical staining using wheat germ agglutinin (WGA), alpha smooth muscle actin (α-SMA), and connexin 43 (CX43) to identify cell membranes, smooth muscle cells, and gap junctions, respectively (Fig. 2A). Using these markers as a guide, we selected spots from each tissue that were identified as coronary resistance microvessels (CRM), perivascular (PV), and myocardium (MYO) subregions (Fig. 2A).

**Fig. 4:**
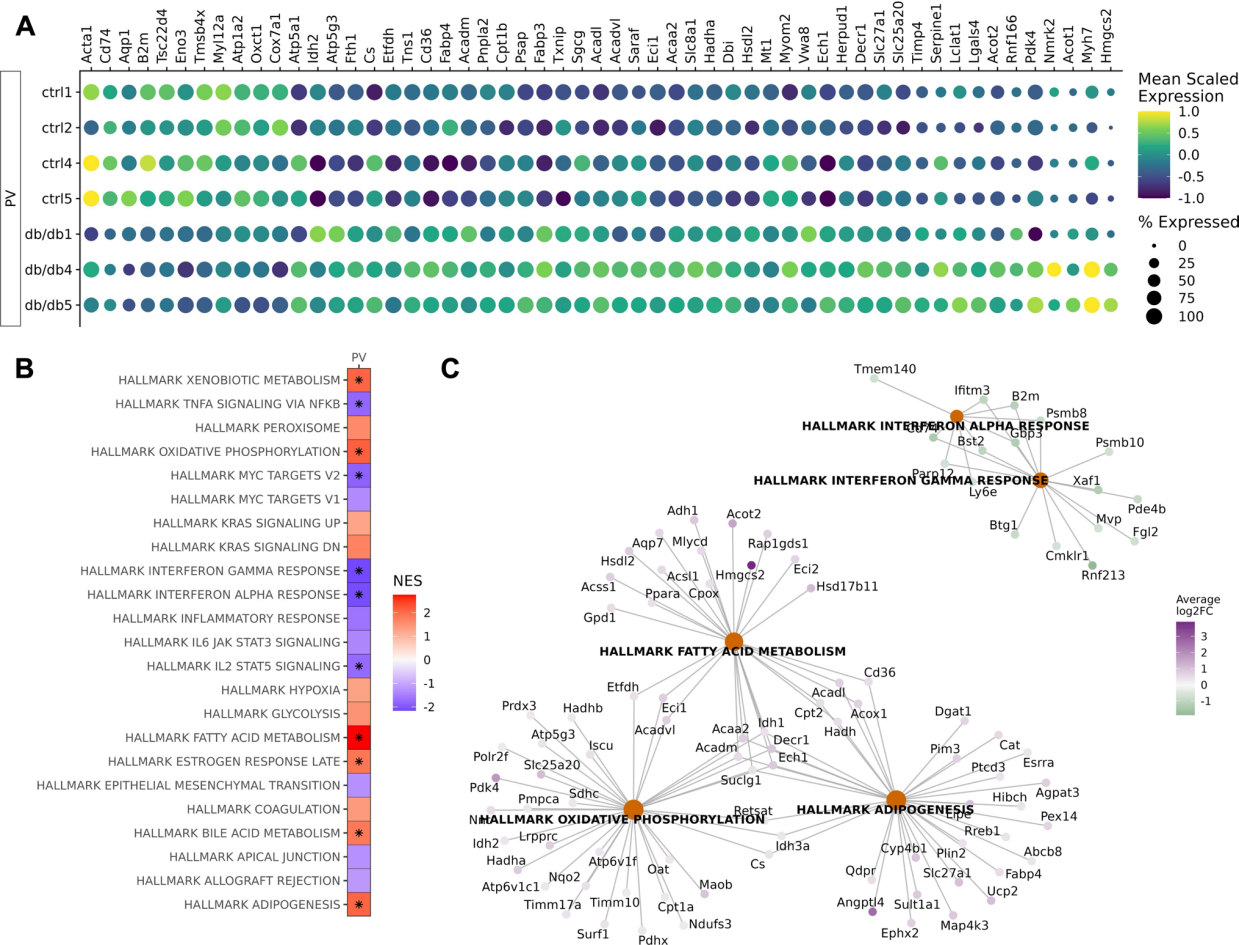
Identification of transcriptomic differences in perivascular (PV) in normal and diabetic hearts. **A** Dot plot depicting significantly differentially expressed genes in PV subregions of the diabetic and normal hearts. **B** Heatmap of Hallmark pathways enriched in differentially expressed genes. **C** Gene concept map of the top five most differentially expressed pathways in the PV subregions. *n* = 3–4 per group

There was a significant increase in *Myh7* transcripts, a marker for cluster 3 *Rnf207* + cardiomyocytes in the CRMs of diabetic mice compared to control (Fig. 2B and Supplemental Table 4), which is consistent with the increased deconvolution weight of cluster 3 *Rnf207* + cardiomyocytes in the diabetic samples. Additionally, the expression of several genes associated with immune cell function was significantly decreased in diabetic CRMs including *B2m*, *C1qc*, *Cd74* (Fig. 2B). Pathway analysis of all differentially expressed genes between control and diabetic CRM subregions revealed significant downregulation in genes associated with interferon alpha and gamma response and significant upregulation of genes associated with xenobiotic metabolism, peroxisome, oxidative phosphorylation, KRAS signaling, fatty acid metabolism, bile acid metabolism, and adipogenesis in diabetic CRMs compared to control (Fig. 2C–D, Table 2, and Supplemental Table 5). In the MYO and PV subregions, we observed a similar decrease in interferon response signatures and upregulation of fatty acid metabolism and adipogenesis genes in diabetic mice compared to control mice (Fig. 3–4 and Supplemental Tables 6–9, respectively). In addition to the interferon response gene signatures, we observed a significant decrease in expression of genes associated with TNF alpha signaling via NFkB, Myc targets, and IL2 STAT5 signaling in diabetic MYO and PV compared to control, suggesting decreased inflammation in the perivascular space (Fig. 4 and Supplemental Table 9).

### Proteomic expression validation

Given that both our single-cell and spatial transcriptomics datasets capture gene expression profiles, we next sought to validate these findings at the protein level by utilizing coronary microvessel proteomics data. Of the top 25 statistically highest spatial CRM gene changes (Supplemental Table 4), 12 proteins followed the exact fold change pattern and were statistically significant in the proteomics dataset (Fig. 2E and Table 3). Only one gene in the top 25 highest spatial CRM gene changes had the opposite expression in the proteomics dataset (Fig. 2E and Table 3), demonstrating that protein expression closely mirrored transcriptomic expression using different methods and datasets.

### Distinct cell–cell interactions in normal and diabetic hearts

**Fig. 5:**
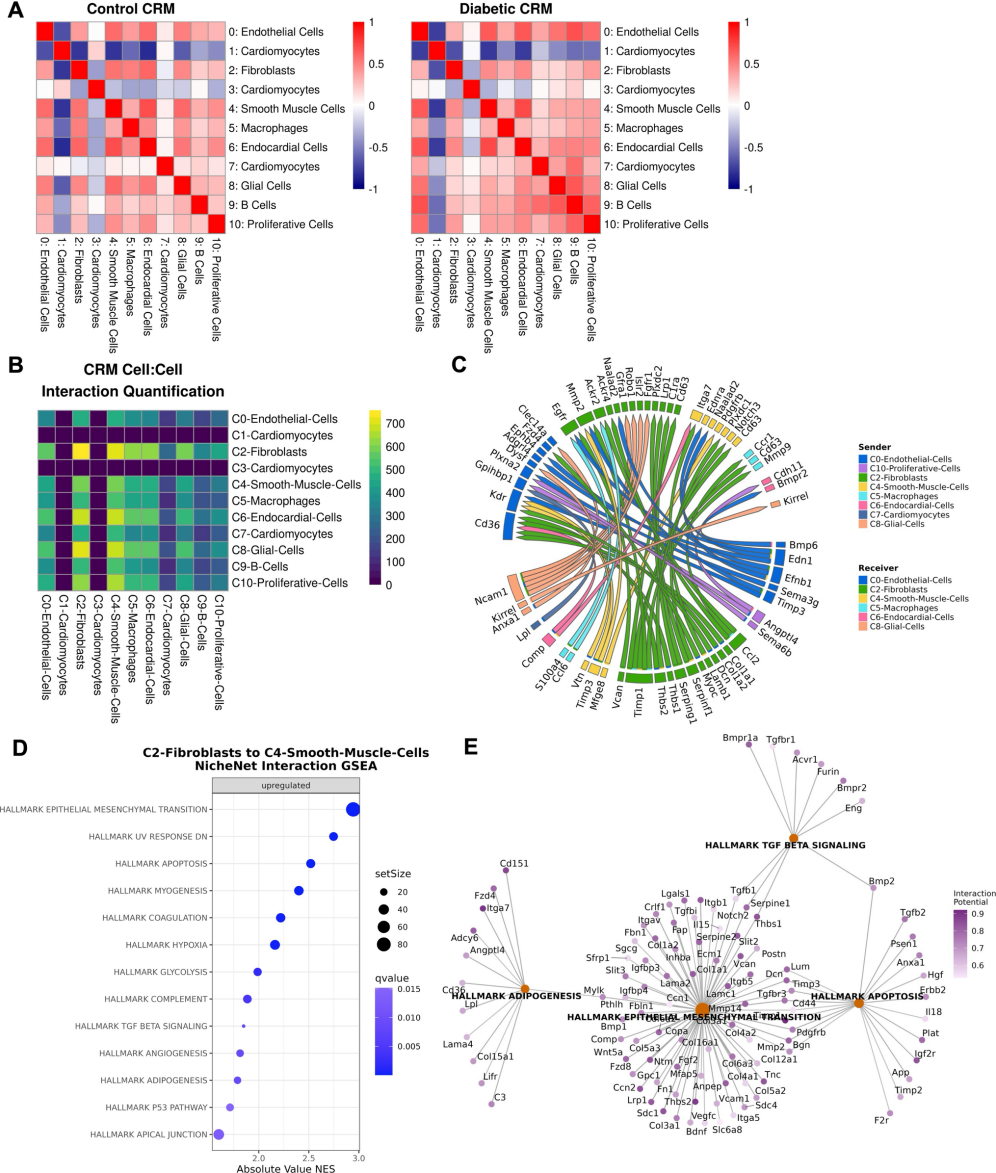
Mapping cell–cell interaction differences in diabetic and normal CRM. **A** Correlation of normalized deconvolution weights of scRNA-seq clusters and cell types in CRM of normal and control hearts. **B** Heatmap depicting the number of high-priority ligand-receptor interactions identified from genes differentially expressed in scRNA-seq clusters and cell types of diabetic and normal hearts. **C** Chord diagram showing top 50 ligand-receptor interactions between scRNA-seq clusters and cell types. **D** Dot plot depicting enrichment of Hallmark pathways in ligand-receptor interactions between C2-Fibroblasts and C4-Smooth-Muscle-Cells. **E** Gene concept network showing interaction potential between C2-Fibroblasts and C4-Smooth-Muscle-Cells for selected pathways. *n* = 1 per group

To assess whether there were differences in the types of cells co-localizing in the CRMs of diabetic and control mice, we calculated the Spearman correlation coefficients for the normalized deconvolution weights calculated from the spatial transcriptomic data in each population. In both control and diabetic mice, cell types typically associated with CRMs including fibroblasts and smooth muscle cells were highly correlated with each other and with endocardial and glial cells, but anti-correlated with cluster 1 *Slc25a4* + cardiomyocytes and cluster 3 *Rnf207* + cardiomyocytes (Fig. 5A; Supplemental Table 10). Using the genes that were differentially expressed between control and diabetic CRM regions, we predicted ligand-receptor pair interactions between frequently co-localizing cell types. We determined that smooth muscle cells and fibroblasts expressed the highest number of receptors interacting with ligands expressed within the CRM (Fig. 5B; Supplemental Table 10). These ligands were frequently expressed in endocardial cells, glial cells, and proliferative cells, as well as fibroblasts and smooth muscle cells themselves (Fig. 5B; Supplemental Table 10). Of the top 50 ligand-receptor pair interactions as ranked by prioritization score, fibroblasts were both the most frequent ligand sender and receiver (Fig. 5C; Supplemental Table 10), demonstrating their critical role in this subregion. As smooth muscle cells were frequently the receivers of ligands from fibroblasts, we sought to better characterize these interactions using gene set enrichment analysis. We show that in diabetic CRM regions, there is an upregulation of ligand-receptor pair interactions from fibroblasts to smooth muscle cells that are associated with pathways such as epithelial to mesenchymal transition, apoptosis, adipogenesis, and TGF beta signaling (Fig. 5D–E).

## Discussion

Cardiovascular disease (CVD) remains the leading cause of mortality in patients with T2D, with CMD emerging as an early and independent complication [12, 33, 63]. Despite its significant contribution to diabetic heart disease, the molecular features associated with CMD remain poorly understood. CMD arises from dysfunction of endothelial and VSMCs, along with remodeling of the walls of the coronary microcirculation [33, 42]. Building upon recent omics studies demonstrating endothelial cell heterogeneity in the heart [50, 58], in this study, we leveraged a novel combination of scRNA-seq and spatial transcriptomics to investigate cellular differences of the coronary microcirculation, perivascular regions, and myocardium in normal and T2D mouse hearts. Our approach utilized fixed single-cell RNA profiling from FFPE and spatial transcriptomics from the same mouse heart tissue that enabled unbiased genomic analysis with spatial resolution, and it allowed for the acquisition of expression data from over 21,000 genes in CRM-enriched spots from specific regions of the mouse heart. Our data unveiled several novel findings pertaining to coronary microcirculation, perivascular regions, and myocardium including an upregulation of genes associated with adipogenesis, fatty acid metabolism, and oxidative phosphorylation in the hearts of T2D mice.

### Metabolic inflexibility and coronary microvascular disease in T2D

CMD is an early and sustained complication of T2D, hypertension, and metabolic syndrome, and it can occur irrespective of occlusive large coronary artery disease. Indeed, the PROMIS-HFpEF study showed that 60–90% of patients with HFpEF have CMD [16]. 30% and 40% of patients with HFpEF also have type 2 diabetes [47]. CMD underlies a myriad of cardiac conditions, and it is an increasingly suspected culprit in the pathogenesis and pathophysiology of heart disease. Fatty acid oxidation is elevated in HFpEF but reduced in heart failure with reduced ejection fraction (HFrEF); however, glucose oxidation is decreased in both HFpEF and HFrEF [15].

A key finding of our study is the extensive transcriptional dysregulation in metabolic pathways within the coronary microcirculation, perivascular regions, and myocardium of T2D mice. The heart predominantly relies on fatty acids (FAs) for ATP production, with metabolic flexibility allowing substrate switching between glucose and FAs based on energy demand. In T2D, however, this adaptability is lost, leading to excessive reliance on FA oxidation, reduced glucose utilization, and mitochondrial dysfunction. This metabolic inflexibility results in an increased production of reactive oxygen species (ROS), lipotoxicity, and ultimately contributes to coronary vascular dysfunction [5, 45, 56, 59].

Consistent with these pathological changes, our scRNA-seq analysis revealed upregulation of genes involved in FA metabolism and oxidative phosphorylation across multiple coronary vascular and cardiac cell populations in diabetic hearts. Notably, hydroxymethylglutaryl CoA synthase 2 (*Hmgcs2)* and acyl-CoA thioesterase 2 (*Acot2)* transcripts were highly expressed in endothelial cells, VSMCs, fibroblasts, and multiple cardiomyocyte clusters. To validate this, we also observed a significantly higher spectral count for *Acot2* in the diabetic CRMs of the proteomics validation set (Table 3); however we did not obtain any spectral hits for *Hmgcs2*. *Hmgcs2* is a ketogenic enzyme that can induce fatty acid β-oxidation and ketogenesis and is regulated by peroxisome proliferator-activated receptor α (PPARα), a fatty acid-activated nuclear receptor that controls metabolic alterations in the liver linked to starvation [66]. In the heart, expression of ventricular *Hmgcs2* increased in both mRNA and protein levels after 4 weeks of high fat diet in mice [28]. Since the heart has limited capacity for true de novo ketogenesis, the function of *Hmgcs2* in the heart is not fully understood [41]. Interestingly, recent beneficial effects of ketones in pathological hypertrophy have been described, potentially via improved myocardial blood flow, although the specific mechanisms were not fully defined [37]. Ketones help prevent coronary microvascular rarefaction [37], which one may conjecture is a potential mechanism to target coronary blood flow improvement given its enrichment in endothelial cells in our current study. These data are intriguing given we have previously reported impaired CBF in db/db mice [30] together with *Hmgcs2* driving diabetic fatty acid metabolism in our current data.

Similarly, *Acot2* transcripts were also highly expressed in diabetic microvascular regions; *Acot2* plays a role in lipid metabolism within the heart by regulating the levels of acyl-CoA, contributing to energy production and membrane synthesis. Hydrolyzing acyl-CoA to free fatty acids and CoA, *Acot2* influences fatty acid availability for oxidation or storage, thus impacting energy balance and metabolic functions in cardiac cells [64]. A key metabolic shift in diabetes is the significant increase in cardiac fatty acid oxidation rates [10]. In rodent models of T2D, increased cardiac fatty acid utilization is linked to decreased cardiac contractile function, which may be linked to lipotoxicity and/or diminished cardiac efficiency [10]. In the diet-induced obesity rodent model, the expression of the ACOT2 protein in the heart and soleus muscle of the rats increased significantly in the high-fat diet group compared to the control diet [17]. In this study, *Acot2* was a key driver of the increased fatty acid metabolism in many of the diabetic cell types via single cell RNA seq. Collectively, metabolic inflexibility and the reliance on FA metabolism, particularly β-oxidation, generate large amounts of ATP, but at the cost of significant oxygen consumption [38]. As such, while CMD can directly contribute to myocardial ischemia [42], the continued heavy reliance on FA metabolism further exacerbates this process in T2D. A previous study showed that increased fatty acid oxidation predominates in db/db mouse hearts beginning at 6 weeks of age, persisting through at least up to 16–18 weeks of age, similar to the present study [3]. That same study showed that this metabolic inflexibility preceded the onset of cardiac dysfunction at 10–12 weeks of age [3]. Other studies have also confirmed cardiac diastolic dysfunction as early as 12 weeks of age in db/db mice [55, 57, 60]. The process of metabolic flexibility/inflexibility relative to the onset of heart failure is complex, but these data may suggest the metabolic inflexibility precedes cardiac dysfunction/HFpEF. Well-controlled studies in preclinical models and humans are warranted to further clarify this timing.

### Oxidative phosphorylation dysregulation in the diabetic coronary microcirculation

Beyond FA metabolism, oxidative phosphorylation pathways were significantly upregulated in diabetic endothelial cells, VSMCs, cardiomyocyte cluster 1, fibroblasts, cardiomyocyte cluster 3, VSMCs, and macrophages. One of the most striking findings was the increased expression of pyruvate dehydrogenase kinase 4 (*Pdk4*); *Pdk4* activity is closely linked to mitochondrial function in VSMCs and plays a crucial role in regulating energy metabolism and metabolic flexibility in the heart by inhibiting the pyruvate dehydrogenase complex, by reducing the conversion of pyruvate to acetyl-CoA [39]. This inhibition shifts the heart's energy metabolism away from glucose oxidation toward fatty acid oxidation [27]. *Pdk4* is so tightly linked to metabolic flexibility that following a single high-fat diet meal, PDK4 cardiac protein and mRNA levels are increased [14]. Interestingly, overnight fasting also causes increased expression of PDK4 in cardiac tissue, implicating its role in the function of maintaining overall glucose homeostatic balance [68]. Clinical studies have demonstrated that PDK4 is upregulated in the calcified vessels of atherosclerotic patients [34]. Treatment with dichloroacetate (DCA), a well-established PDK inhibitor, attenuates atherosclerosis in ApoE^−/−^ western diet-fed mice [49]. These data are intriguing as the db/db mice do not develop atherosclerosis. Further investigation is needed to determine whether this metabolic shift directly contributes to CMD or represents an adaptive response to altered energy demands.

Other mitochondrial transporters and regulators of oxidative phosphorylation, including *Slc25a20* and *Ech1* transcripts, were also highly expressed in diabetic endothelial cells, VSMCs, cardiomyocyte cluster 1, fibroblasts, cardiomyocyte cluster 3, VSMCs, and macrophages. Both Slc25a20 and Ech1 had significantly higher spectral hits in the diabetic CRMs of our proteomics data (Table 3), validating these findings. Slc25a20, also known as the mitochondrial carnitine/acylcarnitine transporter and carnitine acyl-carnitine carrier, plays a crucial role in oxidative phosphorylation and cardiovascular function by facilitating the transport of long-chain acylcarnitines across the mitochondrial membrane, which is essential for the entry of FAs into the mitochondria [29]. By enabling efficient transport and utilization of fatty acids, Slc25a20 supports metabolic flexibility, allowing the heart and other tissues to adapt to varying energy demands and nutrient availability. Overall, there is a void in the literature discussing the cardiovascular role of Slc25a2. One study showed that Slc25a20 is significantly reduced 21 days post-MI in mice infarcted at 7 days old [29]. However, in this study, it appears to play an important role in oxidative phosphorylation in pathophysiologic heart disease in T2D mice. Finally, Ech1, or enoyl-CoA hydratase 1 is crucial for the β-oxidation of fatty acids, a metabolic pathway where long-chain fatty acids are broken down into shorter chains (acetyl-CoA) to produce ATP. As previously mentioned, efficient fatty acid oxidation is vital for meeting the high energy demands of cardiac tissue, especially during increased workload or stress. Additionally, Ech1’s role in fatty acid metabolism helps maintain metabolic flexibility, allowing the heart to adapt to varying energy substrates. Interestingly, Ech1 is significantly reduced in calcified aortic valves and overexpression of Ech1 reduced aortic valve calcification in high cholesterol-fed ApoE^−/−^ mice [54]. In a recent study, Pan et al. postulated that Ech1 may function as a potential diagnostic and therapeutic target for obesity-related CVD [52]. In that study, HFD caused hyperlipidemia and increased oxidative stress, and Ech1 was one of the most significant common cardiac and aortic expressed proteins [52]. In that study, Ech1 was a major contributor to the oxidative phosphorylation pathway in T2D hearts. Together, these findings provide novel insight into transcriptional dysregulation in FA metabolism and oxidative phosphorylation in diabetic hearts. While increased FA oxidation may serve as an early compensatory mechanism, sustained metabolic inflexibility may ultimately promote oxidative stress, mitochondrial dysfunction, and microvascular remodeling. Future studies could investigate whether targeting Pdk4, Slc25a20, or other metabolic regulators can restore substrate flexibility and improve coronary microvascular function in diabetes.

### Adipogenesis is enriched in T2D heart cells

In addition to metabolic inflexibility, we observed significant upregulation of adipogenesis-related genes in diabetic heart cells. Genes such as *Angplt4* and *Ephx2* were highly expressed in endothelial cells, cardiomyocyte cluster 1, fibroblasts, cardiomyocyte cluster 3, VSMCs, and macrophages. Angplt4 plays a crucial role in regulating triglyceride balance and in managing blood vessel permeability, the formation of new blood vessels, inflammatory responses, and is associated with cardiovascular diseases, such as atherosclerosis [31, 62]. Certain members of the Angplt family, particularly Angplt4, play a significant role in the development of obesity, insulin resistance, and diabetes [25]. Peroxisome proliferator‐activated receptors regulate *Angplt4* gene expression, contributing to the modulation of lipid metabolism and insulin sensitivity [25]. In our study, we observed *Angplt4* to be a major contributor to adipogenesis in much of our scRNA-seq data. Another adipogenesis gene, Epoxide hydrolase 2 (*Ephx2*), is the gene that encodes soluble epoxide hydrolase (sEH) that functions to hydrolyze lipid signaling molecules, including the epoxyeicosatrienoic acids (EETs) and epoxidized lipids generated from arachidonic acid [22]. Given this hydrolysis role, sEH is associated with several cardiovascular diseases, including hypertension, cardiac hypertrophy, arteriosclerosis, and heart ischemia/reperfusion injury [26]. sEH is an enzyme that is dysregulated in patients with T2D and is linked to several diabetic complications, particularly in the microvasculature [4]. Treatment with sEH inhibitors reduces cardiotoxicity triggered by hyperglycemia in cardiac cells [40] and protects cardiac myocyte morphology and calcium cycling [20] in T2D. *Ephx2* is a major contributor to adipogenesis in our scRNA-seq data.

### Parallels between scRNA-seq and spatial transcriptomics pathways/genes

Not surprisingly, many of the upregulated pathways in the diabetic cells from the scRNA-seq experiments were also upregulated in diabetic CRM-enriched regions of the spatial transcriptomics data. These included oxidative phosphorylation, fatty acid metabolism, and adipogenesis. Just as in the scRNA-seq data, *Hmgcs2* and *Acot2* seemed to be the key players in fatty acid metabolism in the diabetic CRM-enriched regions, perivascular regions, and myocardium identified by spatial transcriptomics. Overall, disruptions in both oxidative phosphorylation and fatty acid metabolism significantly influence the pathophysiology of diabetes, leading to energy deficits, insulin resistance, and metabolic complications. In keeping with the scRNA-seq data, spatially resolved transcriptomics identified *Pdk4* and *Slc25a20* as key players in oxidative phosphorylation in the diabetic CRM-enriched regions, perivascular regions, and myocardium. Remarkably, *Ucp2* and *Cyp4b1* appeared to be the top drivers of adipogenesis in CRMs by spatial transcriptomics, *Cyp4b1* and *Sult1a1* for myocardium, and *Angplt4* and *Ucp2* for the perivascular regions, whereas *Angplt4* and *Ephx2* were the drivers for the scRNA-seq; however, these genes are still major components of the pathways of the 3 regions in both methods. Intriguingly, in our cell–cell interaction mapping, we show that in diabetic CRM regions, there is an upregulation of ligand-receptor pair interactions from fibroblasts to smooth muscle cells that are associated with pathways including adipogenesis, and of the top 50 ligand-receptor pair interactions as ranked by prioritization score, fibroblasts were both the most frequent ligand sender and receiver. Emerging research suggests that the heart may utilize a classical intercellular lactate shuttle, where lactate is transferred from fibroblasts to cardiomyocytes [19, 67]. Surprisingly, signaling between endothelial cells and VSCMs was not as enriched as between fibroblasts and other cells; the relative signaling using this analysis requires further interrogation to clarify the abundance of signaling to coronary cell types. Oxidative phosphorylation and fatty acid metabolism play crucial roles in diabetes, significantly impacting energy production and metabolic health. Our scRNA-seq and spatial transcriptomics findings collectively support the idea that fatty acid metabolism and oxidative phosphorylation are dysregulated in CRM and the cells that make up the CRMs in diabetic mouse hearts in the absence of atherosclerosis. These data are intriguing and support the well-documented concept that heart failure in T2D patients is led by cardiac metabolic inflexibility, from reduced mitochondrial function, increased reliance on fatty acid oxidation, and impaired glucose utilization resulting in oxidative stress and lipotoxicity [59].

Lastly, this study has a couple of limitations. First, we did not assess whether sex is a biologically significant contributor to variations in the single cell and spatial transcriptomic profiling of the normal and diabetic heart. Future work is needed to address any potential sex differences. Secondly, our myocardial “MYO” clustering was based on regions selected that were not near an observable coronary vessel. Since this was done in a 2-dimensional tissue slice, we are unable to know whether a coronary microvessel was immediately adjacent to the selected region above or below the tissue slice. Nonetheless, the MYO data are intriguing and provide insights into the cardiac transcriptome.

## Conclusions

The findings that we present here based on a novel pairing of scRNA-sequencing and spatial transcriptomics are compelling and reinforce the well-established concept that heart disease in type 2 diabetes is driven in part by metabolic inflexibility in many cardiac cell types, not just myocytes. Utilizing a combination of transcriptomic profiling techniques, our findings provide novel insight into the transcriptional landscape of diabetic CMD, revealing unique expression patterns in the coronary microcirculation in the presence and absence of T2D. We highlight key transcriptional regulators of FA oxidation and oxidative phosphorylation, underscoring their role in metabolic inflexibility leading to oxidative stress, together with adipogenesis, and lipotoxicity. As recently discussed in an editorial, while omics techniques can provide insight into disease pathology, future studies are required to mechanistically dissect causality and potential novel therapeutic options [24]. It will be important to further interrogate these findings in the context of the regulation of CBF and in heart failure. Indeed, perturbations in CBF occur as a cause and consequence of various forms of ischemic and non-ischemic heart failure [23], in some forms owing to coronary vascular origins [36]. CMD is a key driver of metabolic perturbations in the heart in HFpEF [36]. In agreement with this notion, in the present study, db/db mice have demonstrable ischemic HFpEF associated with coronary microvascular disease and impaired CBF, and we identified several upregulated metabolic pathways using our transcriptomics approaches. The novel data presented herein shed light on the underlying single cell and spatial transcriptional landscape that provides evidence for novel pathways to target CMD in heart failure.

## Supplementary Information

Below is the link to the electronic supplementary material.

Supplementary file1 (PPTX 51457 KB)

Supplementary file2 (XLSX 60841 KB)
