# Supplementary material for: Single cell and spatial transcriptomic profiling of the type 2 diabetic coronary microcirculation and myocardium
Source: Basic Res Cardiol. 2025 Nov 7;120(6):1109–29. doi: 10.1007/s00395-025-01144-7 (PMC12680737; doi:10.1007/s00395-025-01144-7)
Supplement: Supplementary file 1 — Supplementary file1 (PPTX 51457 KB) [file 395_2025_1144_MOESM1_ESM.pptx]

## Slide 1
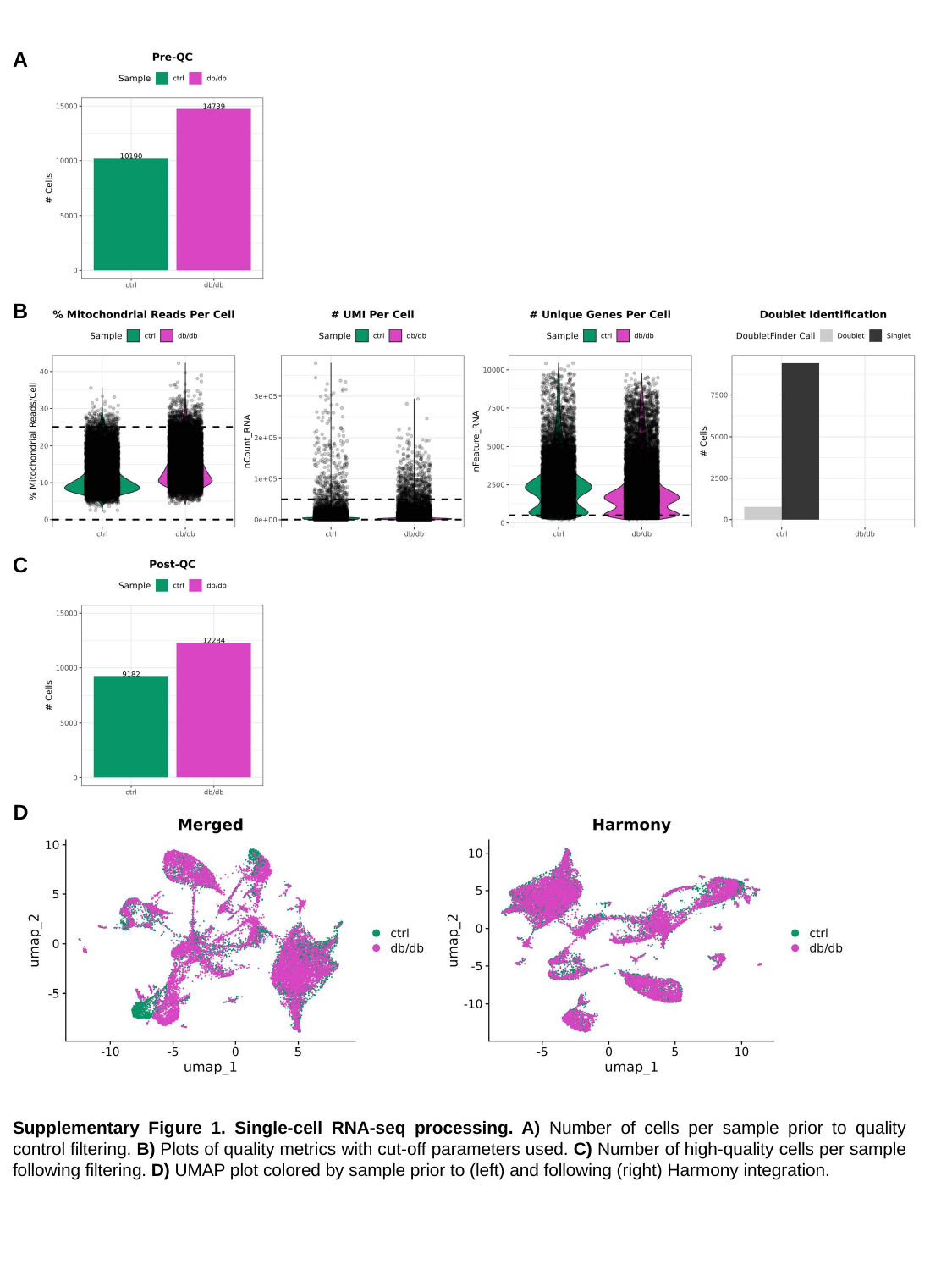

A
B
C
D
Supplementary Figure 1. Single-cell RNA-seq processing. A) Number of cells per sample prior to quality control filtering. B) Plots of quality metrics with cut-off parameters used. C) Number of high-quality cells per sample following filtering. D) UMAP plot colored by sample prior to (left) and following (right) Harmony integration.

## Slide 2
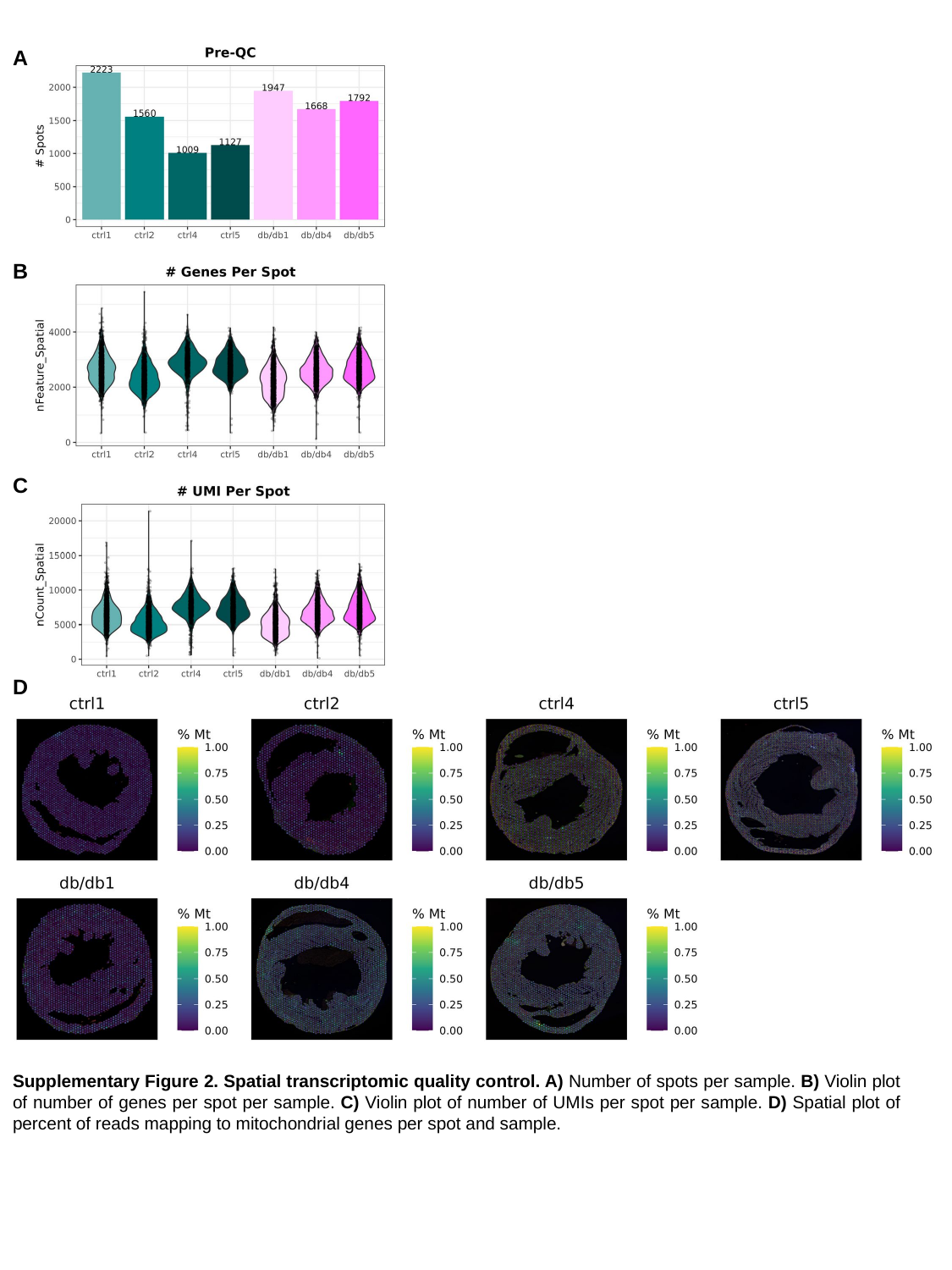

A
B
C
D
Supplementary Figure 2. Spatial transcriptomic quality control. A) Number of spots per sample. B) Violin plot of number of genes per spot per sample. C) Violin plot of number of UMIs per spot per sample. D) Spatial plot of percent of reads mapping to mitochondrial genes per spot and sample.

## Slide 3
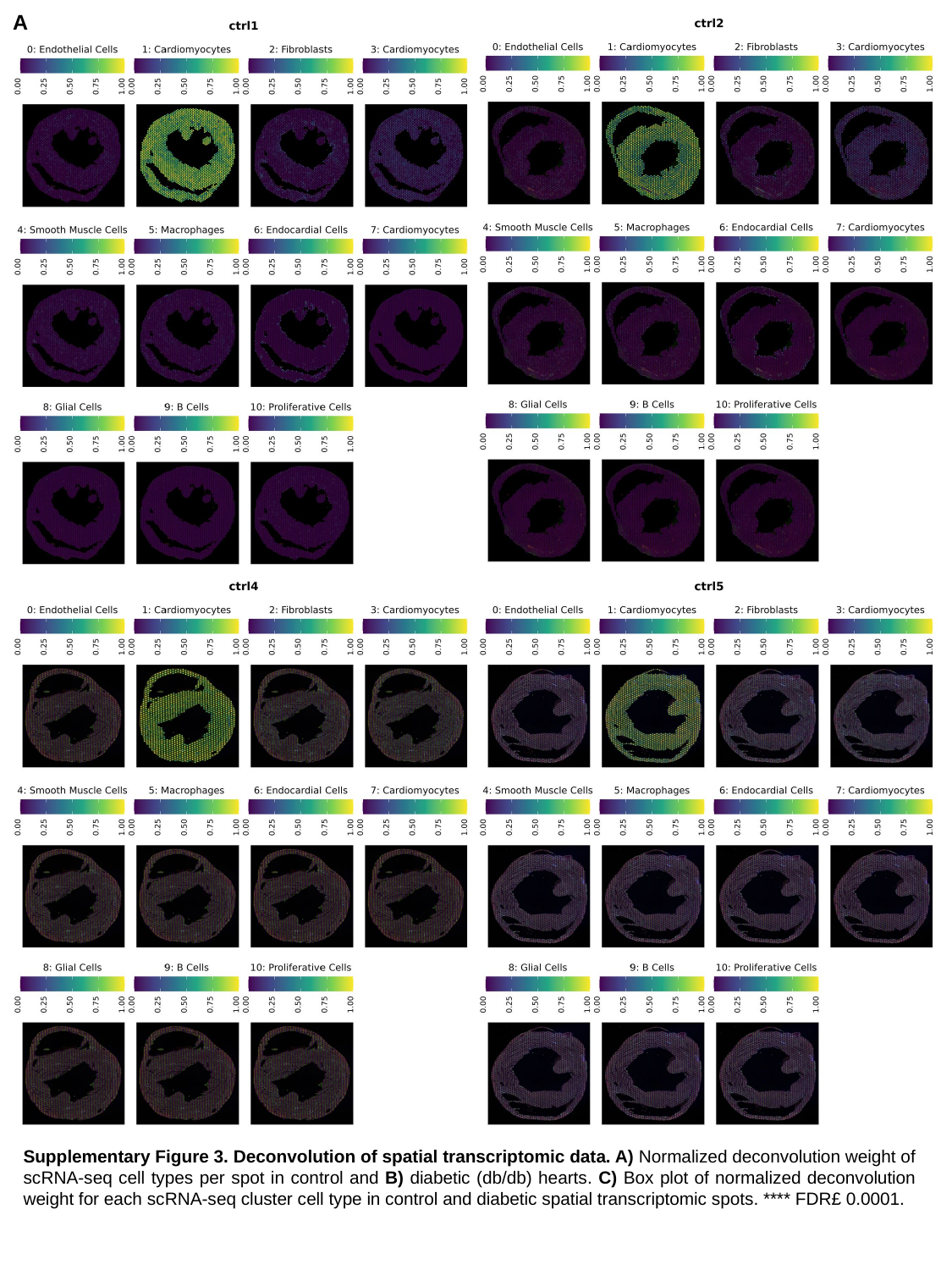

A
Supplementary Figure 3. Deconvolution of spatial transcriptomic data. A) Normalized deconvolution weight of scRNA-seq cell types per spot in control and B) diabetic (db/db) hearts. C) Box plot of normalized deconvolution weight for each scRNA-seq cluster cell type in control and diabetic spatial transcriptomic spots. **** FDR£ 0.0001.

## Slide 4
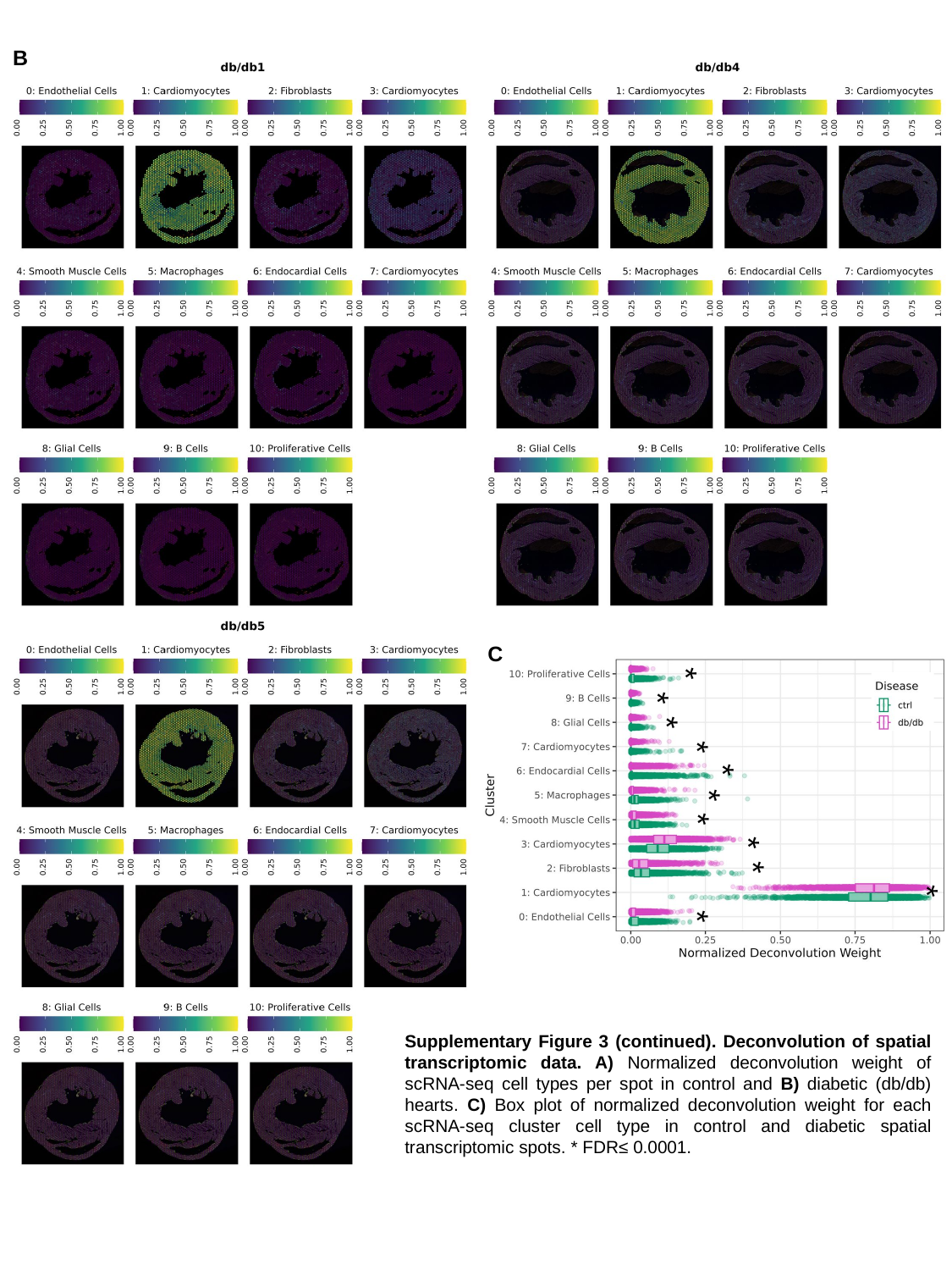

B
C
*
*
*
*
*
*
*
*
*
*
*
Supplementary Figure 3 (continued). Deconvolution of spatial transcriptomic data. A) Normalized deconvolution weight of scRNA-seq cell types per spot in control and B) diabetic (db/db) hearts. C) Box plot of normalized deconvolution weight for each scRNA-seq cluster cell type in control and diabetic spatial transcriptomic spots. * FDR≤ 0.0001.
